# Comparative Analysis of Gastrointestinal Microbiota Along the Digestive Tract in Sika Deer and Reindeer and Prediction of Their Potential Function

**DOI:** 10.3390/ani16101476

**Published:** 2026-05-11

**Authors:** Xinyu Peng, Huansheng Han, Ruihong Hu, Fanzhi Kong, Yuhan Lu

**Affiliations:** 1 College of Animal Science and Technology, Heilongjiang Bayi Agricultural University, Daqing 163319, China; 2 College of Veterinary Medicine, Heilongjiang Agricultural Engineering Vocational College, Harbin 150088, China

**Keywords:** sika deer, reindeer, gastrointestinal microbiota, 16S rRNA, diversity analysis, functional prediction

## Abstract

Gastrointestinal microbiota are essential for fiber degradation, energy metabolism, nutrient utilization, and environmental adaptation in ruminants. Although sika deer and reindeer both belong to the family Cervidae, they differ in ecology, feeding habits, and digestive physiology, which may be reflected in their gastrointestinal microbiota. Here, we used 16S rRNA amplicon sequencing to characterize the gastrointestinal microbial communities of healthy 5-year-old male sika deer and reindeer, with particular attention to taxonomic composition, diversity patterns, and predicted functional profiles across anatomical segments. Both species were dominated by a limited number of highly abundant taxa, but clear interspecific differences were observed. At the phylum level, Bacillota and Bacteroidota predominated in both species, whereas Methanobacteriota was more abundant in reindeer and Bacillota was relatively more abundant in sika deer. At the genus level, methanogen-associated taxa were more prominent in reindeer, whereas *Bacteroides*, Prevotellaceae, and fiber-degradation-related taxa were more abundant in sika deer. Distinct segment-specific microbial patterns were observed along the gastrointestinal tract, especially among the foregut, small intestine, and hindgut. Alpha diversity varied across segments, whereas beta diversity showed clear separation among segments. Functional prediction indicated differences mainly in the overall structure of predicted functional profiles rather than in individual pathways. These findings provide baseline information for understanding gastrointestinal microecology and adaptive mechanisms in cervids.

## Simple Summary

The microbes living in the digestive tract are important for how deer break down plant fiber, absorb nutrients, produce energy, and adapt to different environments. Sika deer and reindeer belong to the same animal family, but they differ in feeding habits and digestive features, which may lead to differences in their gut microbes. In this study, we compared the microbial communities in several digestive tract sections of healthy adult male sika deer and reindeer. We found clear differences both between the two species and among different digestive tract sections. In reindeer, methane-related microbes were more common, while in sika deer, microbes linked to plant fiber breakdown and nutrient use were more abundant. Microbial communities also changed clearly from the foregut to the small intestine and hindgut, showing that digestive tract location strongly influences microbial structure. The predicted roles of these microbes were mainly related to metabolism, especially the use of carbohydrates, amino acids, and energy. These findings provide baseline information that may help improve feeding management, health monitoring, and the understanding of digestive adaptation in deer.

## 1. Introduction

The gastrointestinal microbiota contributes substantially to ruminant nutrition by participating in fiber breakdown, volatile fatty acid production, energy harvest, and maintenance of gut homeostasis [1,2,3]. Compared with monogastric animals, ruminants rely on complex microbial consortia distributed across the foregut and hindgut to convert structurally complex substrates, including crude fiber and agricultural by-products, that are otherwise indigestible to the host, into absorbable and utilizable nutrients [4]. Therefore, variation in microbial composition and functional potential may influence feed utilization, health status, and environmental adaptability. Cervids occupy distinctive ecological niches and possess unique digestive physiological traits, making them valuable models for studying host adaptation and host-microbe interactions. Among cervids, sika deer and reindeer provide an informative comparison because they differ not only in ecological setting, but also in forage use and likely digestive–microbial adaptation. Sika deer are an economically important species in China and are closely associated with nutrient utilization efficiency, velvet antler production, and intensive farming systems, whereas reindeer are adapted to cold northern environments and seasonal forage availability, and can utilize fibrous and lichen-rich diets during periods when high-quality forage is limited [5,6,7]. Reindeer may consume substantial amounts of lichens and other seasonally variable forage resources, and lichen-based feeding has been shown to alter rumen and cecal microbial communities, including methanogenic archaea [5]. In contrast, studies in sika deer indicate that rumen microbial composition and richness are responsive to dietary conditions and forage shifts, suggesting greater dietary flexibility in host–microbiota interactions [6,8]. More broadly, evidence from cervids maintained under the same feeding conditions still supports interspecific differences in intestinal microbiota, while studies across ruminants show that host factors, diet, and the physiological characteristics of different gastrointestinal niches jointly shape microbial colonization and community structure [7,9]. Sika deer and reindeer were selected in this study because they represent two cervid species with distinct economic uses, ecological adaptations, and feeding strategies. Sika deer are one of the major farmed cervids in China, and their economic value is closely associated with velvet antler production, meat production, and intensive deer farming [10,11]. In contrast, reindeer are economically and culturally important animals in northern and Arctic regions, where reindeer husbandry contributes to meat production, hides, antlers, handicrafts, and the livelihoods of local and Indigenous communities [12]. Compared with red deer or elk, sika deer and reindeer show clearer contrasts in ecological and physiological characteristics: sika deer are commonly maintained under intensive or semi-intensive farming systems, whereas reindeer are highly adapted to cold environments and seasonal forage resources. Therefore, comparing these two species provides a useful model for exploring how host ecological adaptation, feeding strategy, and gastrointestinal physiology may be associated with microbial community structure and potential function.

Current studies on cervid gastrointestinal microbiota have mainly focused on single species or discrete anatomical segments, with fecal materials and rumen contents being the most commonly analyzed sample types. Existing studies have shown that different cervid species may still exhibit distinct gut microbial compositions even under the same or similar conditions, and that marked spatial heterogeneity often exists among different gastrointestinal segments within the same species [7,9,13]. Despite these advances, systematic comparative analyses across multiple gastrointestinal segments in sika deer and reindeer remain limited, and the understanding of differences in microbial structure, diversity, and potential function remains incomplete.

In the present study, 16S rRNA sequencing was used to investigate microbial composition, alpha and beta diversity, differentially abundant taxa, and predicted functional profiles across multiple gastrointestinal segments in sika deer and reindeer. This study aimed to clarify species-associated differences and segmental microbial differentiation in cervids, and to provide baseline information for understanding digestive physiology, nutrient utilization, and ecological adaptation in deer.

## 2. Materials and Methods

### 2.1. Experimental Animals and Sample Collection

In this study, three healthy adult male sika deer and three healthy adult male reindeer were selected. All animals were 5 years old and were maintained under comparable feeding and management conditions. The animals were obtained from the Heilongjiang Forest-understory Sika Deer Breeding Base, a farmed cervid breeding facility in Heilongjiang Province, China. The sika deer were farmed animals maintained for velvet antler production, and the reindeer were also farmed animals maintained at the same facility. Because the animals had been raised under human management from an early age, they were accustomed to human contact, which allowed relatively safe handling during sampling. Sampling was conducted on site at the breeding farm in collaboration with farm personnel, whereas subsequent laboratory analyses were performed at Heilongjiang Bayi Agricultural University. Because this study required matched sampling of multiple gastrointestinal segments from healthy adult cervids under comparable management conditions, the number of animals included was limited. Therefore, the present work should be regarded as an exploratory segment-by-segment comparative analysis of the gastrointestinal microbiota.

The animals were maintained within the same area under a combined pen-feeding and grazing system and remained under normal feeding and management conditions before sampling. Feed was withdrawn on the evening before sampling, and gastrointestinal content samples were collected the following morning to minimize the influence of short-term variation in feed intake on microbial composition. Prior to sampling, the animals were first anesthetized outside the enclosure. After they became recumbent and could be safely approached at close range, humane euthanasia was performed by trained personnel via intravenous injection of sodium pentobarbital (Shenyang Congke Chemical Co., Ltd., Shenyang, China), in accordance with the approved animal use protocol of the Ethics Committee of Heilongjiang Bayi Agricultural University (protocol code: DWKJXY2026073). Death was confirmed before sample collection, and every effort was made to minimize handling stress throughout the procedure.

All samples were collected by the same team following a standardized protocol. Sterile gloves, sterile instruments, and sterile cryotubes were used throughout sampling, and instruments were thoroughly disinfected between gastrointestinal segments to prevent cross-contamination and interference from environmental microbes. Samples were immediately frozen in liquid nitrogen after collection and then transferred to a −80 °C freezer until DNA extraction and sequencing analysis.

Sampling covered multiple gastrointestinal segments (Table 1). Specifically, samples from reindeer included the rumen, reticulum, omasum, abomasum, duodenum, jejunum, cecum, colon, and rectum, whereas samples from sika deer included the rumen, reticulum, omasum, abomasum, duodenum, jejunum, ileum, cecum, colon, and rectum. Because ileal samples from reindeer were not successfully retained during the original sampling procedure and could not be retrieved from the liquid-nitrogen sample archive during subsequent sample checking, reindeer ileal contents were not available for DNA extraction and 16S rRNA gene sequencing. Therefore, direct comparisons between reindeer and sika deer were performed only for the gastrointestinal segments shared by both species. The ileal samples from sika deer were included only in the within-species analysis of segmental microbial variation. In total, 27 samples were obtained from reindeer and 30 from sika deer, yielding 57 samples for subsequent 16S rRNA gene sequencing and bioinformatics analysis.

The sample codes used in the figures were defined as follows: X, reindeer; M, sika deer; LW, rumen; WW, reticulum; BW, omasum; ZW, abomasum; SEZC, duodenum; KC, jejunum; HC, ileum; MC, cecum; JC, colon; and ZC, rectum. Ileal samples were available only for sika deer; therefore, the HC group was present only in sika deer.

### 2.2. DNA Extraction, PCR Amplification, and High-Throughput Sequencing

Microbial genomic DNA was isolated from gastrointestinal contents using the E.Z.N.A.^®^ Soil DNA Kit (Omega Bio-Tek, Norcross, GA, USA), following the manufacturer’s protocol. DNA yield and purity were checked with a NanoDrop 2000 spectrophotometer (Thermo Fisher Scientific, Waltham, MA, USA), and DNA integrity was assessed by 1% agarose gel electrophoresis. Samples showing intact bands without obvious degradation were used for PCR amplification.

The V3-V4 region of the bacterial 16S rRNA gene was amplified with primers 341F and 806R using TransStart FastPfu DNA Polymerase (TransGen Biotech, Beijing, China) on an ABI GeneAmp^®^ 9700 thermal cycler (Applied Biosystems, Foster City, CA, USA). Each 20 μL PCR reaction contained 4 μL of 5× FastPfu Buffer, 2 μL of 2.5 mM dNTPs, 0.8 μL of forward primer (5 μM), 0.8 μL of reverse primer (5 μM), 0.4 μL of FastPfu Polymerase, 0.2 μL of BSA, 10 ng of template DNA, and ddH2O to a final volume of 20 μL. 

The PCR cycling conditions were as follows: initial denaturation at 95 °C for 5 min; amplification cycles of 95 °C for 30 s, 58 °C for 30 s, and 72 °C for 45 s; and a final extension at 72 °C for 10 min. For each sample, three PCR replicates were performed and then pooled to reduce amplification bias. Amplicons were checked by 2% agarose gel electrophoresis and purified using the AxyPrep DNA Gel Extraction Kit (Axygen Biosciences, Union City, CA, USA). Purified PCR products were quantified using a Quantus™ Fluorometer (Promega, Madison, WI, USA) and then mixed in appropriate proportions according to the required sequencing depth of each sample.

Sequencing libraries were constructed using the NEBNext® Ultra™ DNA Library Prep Kit (New England Biolabs, Ipswich, MA, USA). High-throughput sequencing was performed on the DNBSEQ-G99 platform (BGI, Shenzhen, China) by Shanghai Biozeron Biotechnology Co., Ltd. (Shanghai, China), using the DNBSEQ-G99RS High-throughput Sequencing Reagent Set (MGI, Shenzhen, China).

### 2.3. Bioinformatic Analysis

Raw reads were processed using fastp v0.20.0 to remove low-quality sequences and reads containing ambiguous bases. Paired-end reads were assembled with FLASH v1.2.7 according to overlap information. After demultiplexing based on barcode and primer sequences, chimeras were removed using Usearch with both de novo and reference-based filtering against the gold database. The retained high-quality sequences were used for subsequent analyses.

An OTU-based analytical strategy was applied. Effective sequences were clustered into OTUs at 97% similarity, and taxonomic assignment was conducted against the SILVA 16S rRNA database v138 using the uclust algorithm. Taxonomic profiles were then summarized from domain to species level.

Downstream analyses were conducted in R v4.0.2 (R Foundation for Statistical Computing, Vienna, Austria). Alpha diversity was assessed using Observed-species, Chao1, Shannon, and Simpson indices. Bray–Curtis and UniFrac distances were calculated for beta-diversity analysis, and PCoA was used to visualize community separation. PERMANOVA, ANOSIM, MRPP, and NMDS were applied to evaluate community differences among groups. Heatmaps and other visualizations were generated using pheatmap package v1.0.12 and ggplot2 package in R v3.5.0, respectively. LEfSe v1.16.0 was used to identify discriminant taxa, and PICRUSt2 v2.5.14 was applied to infer potential microbial functions based on KEGG annotations.

### 2.4. Statistical Analysis

All statistical analyses were performed using the corresponding software platforms. Continuous variables are presented as mean values. Student’s *t*-test was used for direct two-group comparisons of alpha diversity indices between reindeer and sika deer within the same gastrointestinal segment. Because reindeer ileal samples were not available, the ileum was excluded from direct interspecific comparisons. For comparisons among multiple gastrointestinal segments within the same species, one-way analysis of variance (ANOVA) was performed, followed by Tukey’s honestly significant difference (HSD) test. Differences in beta diversity were assessed using PERMANOVA/Adonis based on Bray–Curtis distances, and *p* < 0.05 was considered statistically significant.

## 3. Results

### 3.1. Evaluation of Sequencing Data Quality

A total of 27 samples from reindeer and 30 from sika deer were included in the 16S rRNA high-throughput sequencing analysis (Table 2). In reindeer, the number of effective sequences ranged from 33,244 to 39,030, with a mean of 36,572.52, while the average sequence length ranged from 395.57 to 414.96 bp. In sika deer, the number of effective sequences ranged from 34,097 to 96,381, with a mean of 71,385.03, and the average sequence length ranged from 403.51 to 419.21 bp. The sequencing output and read-length range indicated that the data were suitable for downstream analyses.

### 3.2. Characteristics of Gastrointestinal Microbial Composition

#### 3.2.1. Phylum-Level Composition

**Figure 1:**
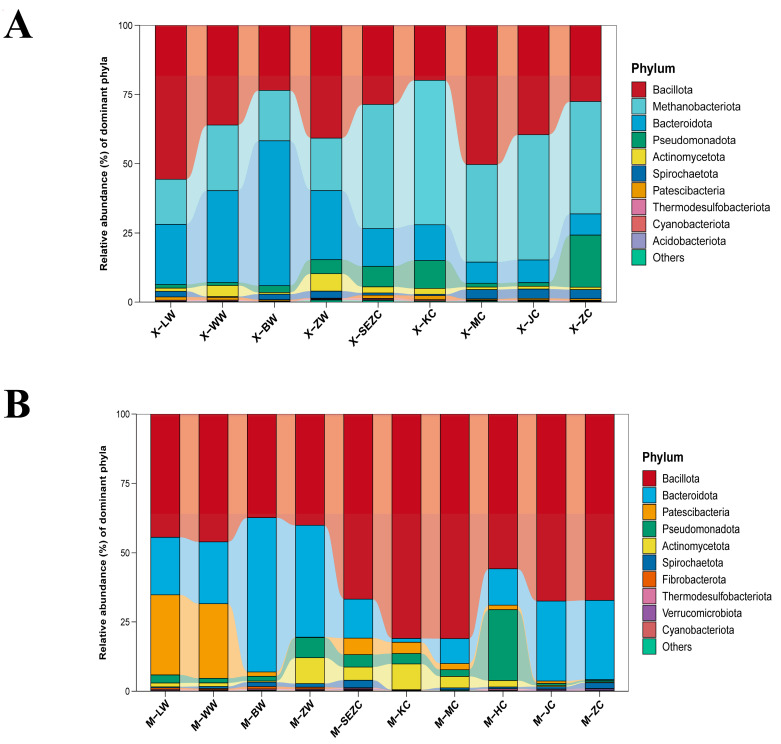
Phylum-level community composition across gastrointestinal segments in reindeer and sika deer. (**A**) Reindeer; (**B**) sika deer. Bars represent the mean relative abundance of dominant bacterial phyla in each gastrointestinal segment. The remaining low-abundance taxa are grouped as “Others”. Sample codes are defined as follows: X, reindeer; M, sika deer; LW, rumen; WW, reticulum; BW, omasum; ZW, abomasum; SEZC, duodenum; KC, jejunum; HC, ileum; MC, cecum; JC, colon; and ZC, rectum. The HC group was present only in sika deer because ileal samples were not available for reindeer.

At the phylum-level analysis (Figure 1), the gastrointestinal microbiota of both reindeer and sika deer were dominated by a limited number of major phyla, although the overall microbial structures differed markedly between the two species. In reindeer, Bacillota, Methanobacteriota, and Bacteroidota constituted the dominant phyla, followed by Pseudomonadota, Actinomycetota, and Spirochaetota. In contrast, sika deer were dominated by Bacillota and Bacteroidota, while Patescibacteria, Pseudomonadota, Actinomycetota, and Spirochaetota also accounted for a certain proportion. Notably, Bacillota was more abundant in sika deer, whereas Methanobacteriota was more prominent in reindeer, indicating clear interspecific differences in methanogen-associated communities.

The phylum-level composition profiles across gastrointestinal segments also revealed marked spatial heterogeneity in microbial structure. In both reindeer and sika deer, the relative abundances of major phyla, including Bacillota, Bacteroidota, Actinomycetota, Spirochaetota, and Pseudomonadota, varied across gastrointestinal segments, indicating clear segment-specific patterns in microbiota composition along the gastrointestinal tract.

#### 3.2.2. Genus-Level Composition

Genus-level profiles showed clear differences in dominant bacterial taxa between reindeer and sika deer. In reindeer, the major genera included *Methanobrevibacter*, Christensenellaceae R-7 group, NK4A214 group, UCG-005, Escherichia-Shigella, Rikenellaceae RC9 gut group, *Xylanibacter*, *Treponema*, Mycoplasmoides, and *Romboutsia*. Among these, *Methanobrevibacter* showed relatively high abundance, which was aligned with the elevated representation of methanogen-associated taxa observed at the phylum level. In sika deer, the dominant genera included *Candidatus Saccharimonas*, Rikenellaceae RC9 gut group, Christensenellaceae R-7 group, *Xylanibacter*, *Ruminococcus*, UCG-005, *Romboutsia*, NK4A214 group, *Clostridium*, *Bifidobacterium*, *Bacteroides*, *Muribaculum*, *Faecalibacterium*, *Monoglobus*, and *Treponema*, as well as several Lachnospiraceae-related genera. Compared with reindeer, Bacteroidota-associated genera, including *Bacteroides*, *Muribaculum*, Prevotellaceae UCG-001, Prevotellaceae UCG-003, and Barnesiella, were more frequently detected in sika deer.

**Figure 2:**
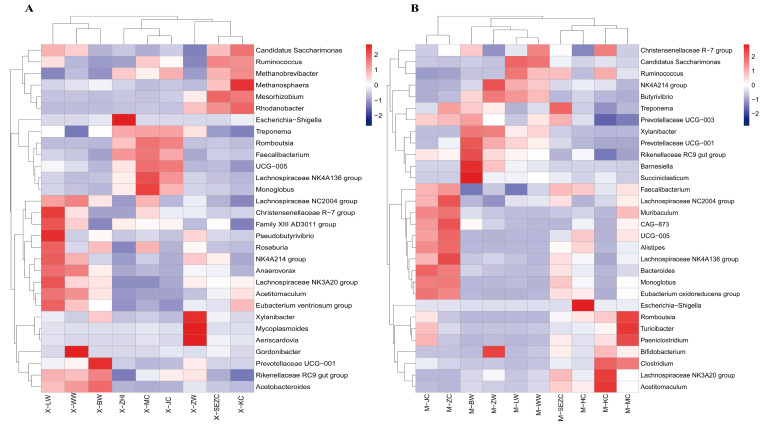
Genus-level heatmap showing segment-specific distribution patterns of dominant bacteria in reindeer and sika deer. (**A**) Reindeer; (**B**) sika deer. Heatmaps are based on the mean relative abundance of dominant genera in each gastrointestinal segment. Colors indicate standardized abundance levels across segments. Sample codes are defined as follows: X, reindeer; M, sika deer; LW, rumen; WW, reticulum; BW, omasum; ZW, abomasum; SEZC, duodenum; KC, jejunum; HC, ileum; MC, cecum; JC, colon; and ZC, rectum. The HC group was present only in sika deer because ileal samples were not available for reindeer.

**Figure 3:**
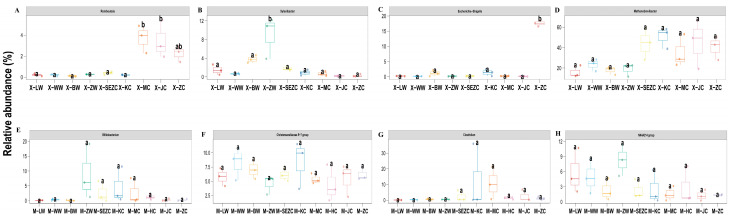
Representative differential genera showing segment-specific abundance patterns in reindeer and sika deer. (**A**–**D**) Representative differential genera in reindeer; (**E**–**H**) representative differential genera in sika deer. Boxes indicate the median and interquartile range, and different lowercase letters indicate significant differences among gastrointestinal segments. Sample codes are defined as follows: X, reindeer; M, sika deer; LW, rumen; WW, reticulum; BW, omasum; ZW, abomasum; SEZC, duodenum; KC, jejunum; HC, ileum; MC, cecum; JC, colon; and ZC, rectum. The HC group was present only in sika deer because ileal samples were not available for reindeer.

The genus-level heatmaps (Figure 2) and the boxplots of representative differential genera (Figure 3) further revealed clear segment-specific distribution patterns of dominant genera along the gastrointestinal tract in both species. In reindeer, Christensenellaceae R-7 group and NK4A214 group were relatively abundant in the foregut, especially in the rumen and reticulum, whereas *Xylanibacter* was relatively enriched in the abomasum. *Methanobrevibacter* showed relatively high abundance in the jejunum, cecum, and rectum, while *Escherichia-Shigella* increased markedly in the rectum. In addition, *Romboutsia* and UCG-005 were more common in the cecum, colon, and rectum. *Treponema* was relatively more abundant in the abomasum and some hindgut segments, suggesting region-specific differences in substrate utilization.

In sika deer, *Candidatus Saccharimonas* was relatively abundant in the rumen and reticulum, indicating a pronounced foregut enrichment pattern. The Christensenellaceae R-7 group was generally more abundant in foregut segments and less abundant in the hindgut. *Xylanibacter*, UCG-005, and certain Prevotellaceae UCG-003-related taxa were relatively enriched in the omasum and abomasum, and *Bifidobacterium* showed relatively high abundance in the omasum. By contrast, *Muribaculum*, *Romboutsia*, *Clostridium*, *Turicibacter*, and *Bacteroides* were more common in hindgut segments, including the cecum, colon, and rectum, suggesting a greater ecological advantage in the hindgut niche.

Overall, both reindeer and sika deer exhibited clear genus-level segmentation among the foregut, small intestine, and hindgut. However, the combinations of dominant genera and their segment-specific enrichment patterns differed between the two species. Reindeer were characterized by a relatively greater prominence of methanogen-associated genera, particularly *Methanobrevibacter*, whereas sika deer showed a higher prevalence of *Bacteroides*, *Muribaculum*, and several Prevotellaceae-related taxa in specific foregut or hindgut segments. These findings suggest that both host species and gastrointestinal segments contribute to the formation of structured microecological patterns.

### 3.3. Alpha Diversity Analysis of the Microbiota

**Figure 4:**
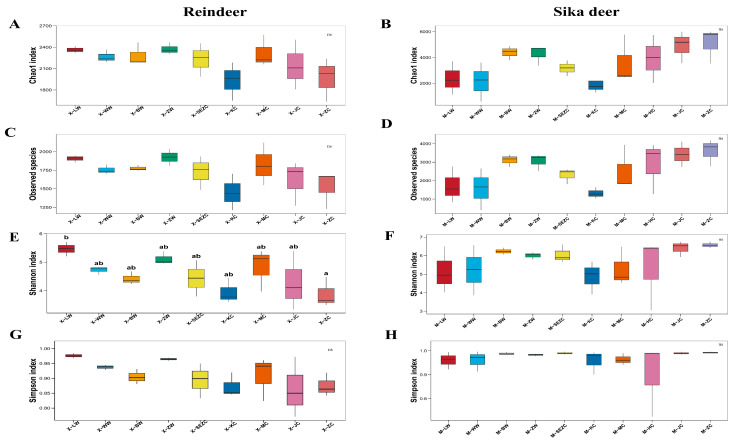
Alpha diversity indices of gastrointestinal microbiota across different segments in reindeer and sika deer. (**A**,**C**,**E**,**G**) Chao1, Observed-species, Shannon, and Simpson indices in reindeer, respectively; (**B**,**D**,**F**,**H**) the corresponding indices in sika deer. Boxes indicate the median and interquartile range, and points represent biological replicates. Different lowercase letters indicate significant differences among gastrointestinal segments based on one-way ANOVA followed by Tukey’s HSD test, whereas “ns” indicates no significant difference. Sample codes are defined as follows: X, reindeer; M, sika deer; LW, rumen; WW, reticulum; BW, omasum; ZW, abomasum; SEZC, duodenum; KC, jejunum; HC, ileum; MC, cecum; JC, colon; and ZC, rectum. The HC group was present only in sika deer because ileal samples were not available for reindeer.

Alpha diversity indices are shown in Figure 4, and detailed numerical values are provided in Supplementary Table S1. Overall, the richness and diversity of microbiota differed to some extent among gastrointestinal segments in both reindeer and sika deer. In reindeer, the Shannon index was relatively high in the rumen and abomasum but relatively low in the jejunum and rectum, with Observed-species displaying a similar trend. In sika deer, the Shannon index was relatively high in the rectum and colon, followed by the omasum and duodenum, whereas it was relatively low in the jejunum; Observed-species was also relatively elevated in the rectum, colon, and omasum. In terms of mean values, alpha diversity indices in most gastrointestinal segments of sika deer were higher than those in reindeer.

Despite these numerical patterns, statistical comparisons indicated that relatively few differences reached significance. In reindeer, significant differences in the Shannon index were detected only between a limited number of segments, indicating some degree of differentiation in microbial diversity among segments. In sika deer, no significant differences were observed among segments for Observed-species, Chao1, Shannon, or Simpson, suggesting that microbial richness and diversity were relatively stable overall and that segmental differentiation was not pronounced. Taken together, alpha diversity analysis indicated that microbial richness and evenness varied to some extent across gastrointestinal segments in both species, but these differences were manifested primarily as numerical fluctuations rather than widespread, statistically significant changes.

For direct interspecific comparisons, Student’s t-test was performed to compare alpha diversity indices between reindeer and sika deer within each shared gastrointestinal segment. Because reindeer ileal samples were not available, the ileum was excluded from this analysis. The detailed pairwise comparison results are provided in Supplementary Table S2. Overall, alpha diversity indices were numerically higher in sika deer than in reindeer in several shared segments, particularly in the omasum, abomasum, colon, and rectum, whereas differences in the rumen, reticulum, jejunum, and cecum were generally not significant. Significant differences were observed for all four alpha diversity indices in the omasum and abomasum, and for most indices in the colon and rectum.

### 3.4. Beta Diversity Analysis of the Microbiota

**Figure 5:**
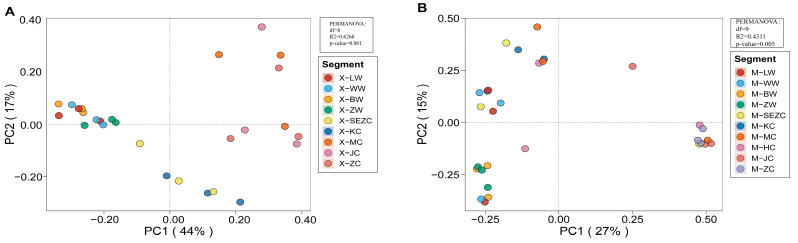
Principal coordinate analysis (PCoA) of beta diversity across gastrointestinal segments in reindeer and sika deer. (**A**) Reindeer; (**B**) sika deer. Each point represents one sample, and colors indicate gastrointestinal segments. Insets show the PERMANOVA results for overall community differences among segments. Sample codes are defined as follows: X, reindeer; M, sika deer; LW, rumen; WW, reticulum; BW, omasum; ZW, abomasum; SEZC, duodenum; KC, jejunum; HC, ileum; MC, cecum; JC, colon; and ZC, rectum. The HC group was present only in sika deer because ileal samples were not available for reindeer.

Beta diversity patterns are shown in the PCoA plots (Figure 5) and Table 3. PCoA revealed clear separation of microbial community structures among different gastrointestinal segments in both reindeer and sika deer. Samples from the same segment tended to cluster together, whereas samples from different segments showed distinct distributions. In reindeer, PC1, PC2, and PC3 explained 44%, 17%, and 10% of the community variation, respectively. The PCoA pattern indicated clear separation between foregut and hindgut samples, while small-intestinal samples were positioned intermediately, thereby reflecting a gradual structural transition along the gastrointestinal tract. In sika deer, PC1, PC2, and PC3 explained 27%, 15%, and 10% of the variation, respectively. Samples from different segments also showed a clustering tendency, and clear distributional differences were observed among foregut, small-intestinal, and hindgut samples, although some overlap remained between adjacent segments.

PERMANOVA analysis (Table 3) supported these observations by demonstrating statistically significant differences in microbial community structure among gastrointestinal segments in both reindeer (R^2^ = 0.62682, F = 3.7793, *p* = 0.001) and sika deer (R^2^ = 0.4311, F = 1.684, *p* = 0.005). The relatively higher R^2^ value observed in reindeer may indicate that the gastrointestinal segment accounts for a larger proportion of community variation in this species compared with sika deer.

Taken together, the PCoA patterns and between-group statistical tests indicate that segmental differentiation of the gastrointestinal microbiota was generally stronger in reindeer than in sika deer, especially between the foregut and hindgut and between some small-intestinal and hindgut segments. Sika deer also showed replacement of community structure from the foregut to the hindgut, but some overlap persisted between adjacent segments. Overall, beta diversity was more sensitive than alpha diversity in revealing structural differentiation of the microbiota among gastrointestinal segments.

### 3.5. Differential Taxa Across Gastrointestinal Segments and Between Species

Differential taxon analysis (Figure 3) showed marked differences in the dominant microbiota across gastrointestinal segments between reindeer and sika deer, mainly reflected in shifts in the relative abundance of a limited number of core genera. In reindeer, genera such as *Methanobrevibacter*, *Escherichia-Shigella*, *Romboutsia*, *Monoglobus*, and *Xylanibacter* showed obvious abundance differences among segments. In sika deer, *Candidatus Saccharimonas*, *Muribaculum*, *Bacteroides*, *Clostridium*, and *Turicibacter* also exhibited strong segmental differentiation.

In reindeer, the Christensenellaceae R-7 group, NK4A214 group, *Methanobrevibacter*, *Escherichia-Shigella*, *Monoglobus*, *Romboutsia*, *Treponema*, UCG-005, and *Xylanibacter* displayed varying degrees of intersegmental differences. The Christensenellaceae R-7 group and NK4A214 group were relatively abundant in foregut segments, suggesting adaptation to the foregut fermentation environment. *Escherichia-Shigella* increased markedly in the rectum, showing clear enrichment at the distal end of the intestine. *Monoglobus* and *Romboutsia* were more common in hindgut segments, including the cecum, colon, and rectum. *Methanobrevibacter* showed relatively high abundance in the abomasum and some hindgut segments, highlighting strong segmental differentiation of methanogen-associated communities along the reindeer gastrointestinal tract. Overall, differential taxa in reindeer mainly displayed stratified distributions among the foregut, small intestine, and hindgut, with the greatest contrast occurring between the foregut and hindgut.

In sika deer, *Candidatus Saccharimonas*, Christensenellaceae R-7 group, *Bifidobacterium*, *Muribaculum*, *Romboutsia*, *Clostridium*, *Turicibacter*, UCG-005, *Xylanibacter*, and some Prevotellaceae-related taxa also showed significant differences among gastrointestinal segments. Specifically, *Candidatus Saccharimonas* was relatively abundant in the rumen and reticulum, showing a tendency toward foregut enrichment. Prevotellaceae UCG-003, *Xylanibacter*, and some UCG-005-related taxa were relatively more abundant in the omasum and abomasum. By contrast, *Muribaculum*, *Romboutsia*, *Clostridium*, *Turicibacter*, and *Bacteroides* were more widely distributed in hindgut segments such as the cecum, colon, and rectum, suggesting their roles in continued fermentation and utilization of residual substrates in the hindgut. In addition, *Bifidobacterium* and some Christensenellaceae R-7 group taxa showed relatively high abundance in certain small-intestinal or foregut segments, indicating obvious spatial partitioning among the foregut, small intestine, and hindgut in sika deer as well.

For direct interspecific comparison, only the nine gastrointestinal segments shared by reindeer and sika deer were included because reindeer ileal samples were not available. Across these shared segments, reindeer showed a relatively higher abundance of methanogen-associated taxa, especially *Methanobrevibacter*, whereas sika deer showed higher representation of several Bacteroidota-related and polysaccharide-utilization-associated genera, including *Bacteroides*, *Muribaculum*, and several Prevotellaceae-related taxa. These differences were particularly evident in the foregut and hindgut segments, suggesting that reindeer and sika deer may differ in gastrointestinal fermentation patterns and substrate utilization strategies.

Comparison of differential taxa between the two species showed that methanogen-associated genera such as *Methanobrevibacter* were more prominent in reindeer, whereas *Bacteroides*, *Muribaculum*, *Turicibacter*, *Clostridium*, and some Prevotellaceae-related taxa displayed more obvious segment-specific variation in sika deer. This suggests that reindeer and sika deer differ to some extent in gastrointestinal microecological composition, substrate utilization patterns, and potential fermentation modes. Overall, differential taxon analysis revealed that both host species identity and gastrointestinal location contribute to the organization of distinct yet structured microecological communities.

LEfSe analysis further identified segment-specific taxa within each species. In sika deer, differentially abundant taxa were mainly enriched in the rumen, reticulum, omasum, abomasum, and duodenum, including Patescibacteria-related taxa, Prevotellaceae, *Alloprevotella*, *Parabacteroides*, and Lachnospiraceae UCG-008. In reindeer, a larger number of differentially abundant taxa were detected among gastrointestinal segments, suggesting stronger segmental differentiation of the microbiota. Detailed LEfSe results are provided in Supplementary Table S3. The detailed LEfSe results are provided in Supplementary Table S3, which has been uploaded separately due to its large size.

### 3.6. Prediction of Potential Functions of the Microbiota

KEGG level 2 functional annotation showed that (Table 4) the predicted microbial functions in both species were concentrated mainly in metabolic pathways, genetic information processing, and environmental information processing. The most represented categories included carbohydrate metabolism, amino acid metabolism, energy metabolism, membrane transport, translation, and nucleotide metabolism. Reindeer showed relatively higher proportions of energy metabolism, translation, and nucleotide metabolism than sika deer, suggesting possible species-related differences in functional allocation within the microbial communities.

**Figure 6:**
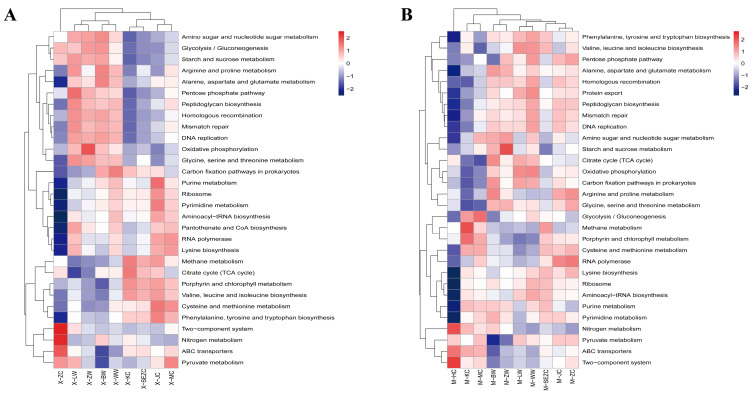
Sample-level KEGG level 3 heatmap of predicted functions. (**A**) The heatmap shows the predicted functional abundance of KEGG level 3 pathways across individual gastrointestinal samples from reindeer. (**B**) The heatmap shows the predicted functional abundance of KEGG level 3 pathways across individual gastrointestinal samples from sika deer. Sample codes are defined as follows: X, reindeer; M, sika deer; LW, rumen; WW, reticulum; BW, omasum; ZW, abomasum; SEZC, duodenum; KC, jejunum; HC, ileum; MC, cecum; JC, colon; and ZC, rectum. The HC group was present only in sika deer because ileal samples were not available for reindeer.

At KEGG level 3 (Figure 6), heatmap-based clustering revealed segment-associated variations in predicted functional profiles in both species. Across different gastrointestinal segments of sika deer, notable variations were observed in several pathways, including glycolysis/gluconeogenesis, amino sugar and nucleotide sugar metabolism, starch and sucrose metabolism, methane metabolism, ABC transporters, two-component systems, ribosome, and pyruvate metabolism. In reindeer, several pathways showed stronger segmental differentiation, namely methane metabolism, the citrate cycle (TCA cycle), carbon fixation pathways in prokaryotes, glycolysis/gluconeogenesis, ABC transporters, two-component systems, and pyruvate metabolism. When the predicted functional profiles were compared between species, reindeer showed relatively higher proportions of energy metabolism, translation, and nucleotide metabolism, whereas sika deer showed relatively higher proportions of carbohydrate metabolism and lipid metabolism. These functional differences correspond to the taxonomic patterns described above, although they should be interpreted as predicted functional potential rather than direct metagenomic evidence.

PERMANOVA analysis of the overall functional profiles (Table 5) indicated significant differences among gastrointestinal segments in both sika deer (R^2^ = 0.49943, *p* = 0.004) and reindeer (R^2^ = 0.46203, *p* = 0.040), indicating that gastrointestinal location significantly influenced the predicted functional profiles of the microbiota in both species. Combined with the ANOSIM results, functional stratification across segments appeared relatively stable in sika deer (R = 0.2892, *p* = 0.001), whereas segmental differences in reindeer were present but did not reach significance in ANOSIM (R = 0.1433, *p* = 0.075). However, when individual KEGG pathways were tested separately, no pathway at KEGG level 1 or level 3 reached the conventional significance threshold (*p* < 0.05) in either reindeer or sika deer. In other words, functional differences among gastrointestinal segments in both species were reflected more in changes in the overall structure and clustering pattern of the predicted functional profile than in significant shifts in any single pathway.

## 4. Discussion

### 4.1. Differences in Gastrointestinal Microbiota Composition Between Sika Deer and Reindeer and Possible Causes

This study demonstrated that gastrointestinal microbiota communities differ between sika deer and reindeer across multiple anatomical segments, with divergence evident at both the phylum and genus levels. At the phylum level, Bacillota and Bacteroidota were the main dominant phyla in sika deer, whereas reindeer showed relatively high abundance of Methanobacteriota in addition to Bacillota and Bacteroidota. At the genus level, *Methanobrevibacter* was more prominent in reindeer, whereas *Bacteroides* and Prevotellaceae-related taxa were relatively more abundant in sika deer. These patterns indicate that, despite their shared classification as cervid ruminants, the two species harbor microbiota that retain discernible host-specific features.

Such differences may be interpreted in relation to host-associated factors, including genetic background, gastrointestinal physicochemical conditions, digestive physiology, digesta transit time, and local redox status, all of which have been reported to contribute to microbial colonization and community stability [9,14,15]. Although sika deer and reindeer belong to the same family, they differ in ecological adaptation, feeding structure, and energy utilization strategy, and these differences may be reflected in the construction of gastrointestinal microbial niches [16,17].

In addition, the relatively high abundance of Methanobacteriota and its representative genus *Methanobrevibacter* in reindeer suggests that hydrogenotrophic methanogenic archaea may be more abundant in the reindeer gastrointestinal tract. Methanogenic archaea are known to utilize H2 and CO2 generated during microbial fermentation, thereby contributing to redox balance and indirectly influencing fiber degradation efficiency and volatile fatty acid production patterns [5,18]. Therefore, enrichment of methanogen-associated taxa in reindeer may reflect a microecological adaptation strategy for energy metabolism and fermentation regulation that differs from that of sika deer.

In contrast, the higher prevalence of *Bacteroides*, Prevotellaceae-related taxa, and certain Lachnospiraceae-associated genera in sika deer may indicate a community structure more strongly oriented toward the degradation of polysaccharides, proteins, and complex plant substrates [19,20]. This suggests that the gastrointestinal microbiota of sika deer may have greater potential to utilize carbohydrates and complex organic substrates, consistent with the higher relative abundance of functional categories, such as carbohydrate and lipid metabolism, predicted for sika deer in the present study [21]. The correspondence between taxonomic composition and predicted function should be interpreted with caution, given PICRUSt2’s inferential nature; the observed alignment suggests that differences in microbial structure may be linked to distinct substrate utilization strategies between the two species.

### 4.2. Patterns of Microbial Differentiation Across Gastrointestinal Segments and Their Biological Significance

This study found clear spatial heterogeneity in the microbiota among gastrointestinal segments in both reindeer and sika deer. At both the phylum and genus levels, microbial communities exhibited segment-specific distributions, with samples from the same anatomical region clustering, whereas those from different segments were more clearly separated. Beta-diversity analyses further confirmed that the gastrointestinal segment is a major source of variation in microbial community structure in both species.

These results are consistent with the basic physiological pattern of the ruminant gastrointestinal tract, often described as foregut fermentation, small-intestinal digestion and absorption, and hindgut re-fermentation [21,22]. Different gastrointestinal regions differ substantially in fermentation substrates, pH, microbial colonization patterns, and nutrient transport modes, so it is expected that different segments form different microbial structures [23]. In the present study, foregut segments in both reindeer and sika deer were enriched with taxa that have been reported to be associated with fiber degradation and anaerobic fermentation, whereas the microbial patterns observed in the small intestine and hindgut may be related to nutrient absorption, bile salt exposure, and continued fermentation of residual substrates. These functional features were inferred from the combined interpretation of taxonomic composition, segment-specific differential taxa, PICRUSt2-based KEGG functional prediction, and previously reported ecological roles of these microbial groups in ruminant gastrointestinal tracts. For example, taxa enriched in the foregut were interpreted in relation to their reported associations with fiber degradation and anaerobic fermentation, whereas microbial patterns in the small intestine and hindgut were interpreted together with the known physiological characteristics of these segments, including bile acid exposure, nutrient absorption, and residual substrate fermentation. Therefore, these interpretations should be regarded as inferred microbial functional potential rather than direct evidence from metagenomic, transcriptomic, or metabolomic measurements. This is consistent with the functional division of labor among different gastrointestinal segments in ruminants.

Notably, the small-intestinal microbiota often showed strong separation from the foregut microbiota in community structure. This may be related to higher bile acid concentration, enhanced digestive enzyme activity, reduced availability of fermentable substrates, and faster digesta flow in the small intestine, all of which exert stronger selective pressure on the microbial community [24,25]. In contrast, the cecum, colon, and rectum are more likely to enrich taxa involved in re-fermentation of residual substrates, short-chain fatty acid production, and mucosal interaction. Therefore, hindgut segments often exhibit both similarity and local differentiation [26,27].

### 4.3. Ecological Interpretation of Alpha and Beta Diversity

From the perspective of alpha diversity, Shannon and Observed-species exhibited variations across gastrointestinal segments in both reindeer and sika deer, but relatively few comparisons reached statistical significance. In contrast, beta diversity analyses more clearly captured the structural separation of microbial communities across segments.

This pattern suggests that, under the conditions of this study, the main differences among gastrointestinal segments are more likely reflected in turnover of community composition than in abrupt changes in overall diversity [9,13]. In other words, segments may harbor comparable levels of diversity while differing in the identities and relative abundances of their constituent taxa. This is one reason why beta diversity is often more sensitive than alpha diversity. Such observations are consistent with the notion that gastrointestinal regions are differentiated less by the quantity of microbial taxa present than by their functional and ecological composition [28].

In addition, mean alpha diversity values were generally higher in sika deer than in reindeer across corresponding gastrointestinal segments. This may imply that the available substrates in the gastrointestinal segments of sika deer are more diverse, or that the host’s internal environment can accommodate a wider range of microbial groups [29]. It should be noted that the number of effective sequences was higher overall in sika deer than in reindeer, suggesting differences in sequencing depth between the species. However, raw sequence count mainly reflects sequencing yield rather than microbial richness itself. Therefore, interpretations of richness and diversity in this study were based primarily on alpha diversity metrics such as Observed-species, Chao1, and Shannon rather than on raw read counts. The generally higher alpha diversity observed across most sika deer segments may be associated with host substrate utilization, local microenvironmental differences, and the detectability of low-abundance taxa, but this trend still requires validation using standardized analyses and larger sample sizes [30,31].

### 4.4. Differential Taxa Between Species and Their Potential Functions

Differential taxon analysis in this study showed that the dominant microbiota differed markedly among gastrointestinal segments in reindeer and sika deer, and that the major dominant genera exhibited distinct abundance patterns across segments. Intergroup comparisons of the top 10 dominant genera showed that *Methanobrevibacter*, *Treponema*, *Romboutsia*, UCG-005, and *Xylanibacter* varied markedly among segments in reindeer, whereas *Bifidobacterium*, *Candidatus Saccharimonas*, *Muribaculum*, *Romboutsia*, *Xylanibacter*, and some Prevotellaceae-related taxa showed strong segmental differentiation in sika deer. These findings indicate that the main differences between the two species lie in the segmental distribution patterns and relative abundance levels of core dominant genera.

The biological implications of these taxa warrant careful consideration. For example, *Treponema*, *Xylanibacter*, *Ruminococcus*, Prevotellaceae, and Lachnospiraceae-related taxa are often associated with the degradation and utilization of hemicellulose, cellulose, and other complex polysaccharides and are important members of foregut fermentation communities in ruminants [19,32,33]. In contrast, certain Bacillota members, such as *Faecalibacterium*, *Roseburia*, and *Eubacterium*, have been linked to the production of short-chain fatty acids, particularly butyrate, which may contribute to intestinal ecological stability and host mucosal health [34,35].

The enrichment of methanogenic archaea and related functional groups in the reindeer gastrointestinal tract suggests that its fermentation system may rely more heavily on hydrogen metabolism and methane production to maintain metabolic balance [5]. By contrast, the greater representation of Bacteroidota and certain groups associated with lactic acid bacteria in sika deer may indicate an alternative microecological configuration oriented toward the degradation of complex substrates, the utilization of oligosaccharides, and adaptation to localized acidic environments [36,37]. Therefore, differential taxa reflect not only segmental differences but also distinct host strategies in nutrient utilization, energy metabolism, and environmental adaptation.

The LEfSe results further supported the segment-specific distribution patterns of dominant taxa, indicating that microbial differences in both species were not only reflected in relative abundance changes in dominant genera but also in statistically identified differential taxa.

### 4.5. Potential Functions of the Microbiota

Functional prediction based on PICRUSt2 indicated that, in both species, microbial communities across gastrointestinal segments were predominantly enriched in pathways associated with metabolism, genetic information processing, and environmental information processing, with particular emphasis on carbohydrate, amino acid, energy, membrane transport, translation, and nucleotide metabolism. This is consistent with the general characteristics of gastrointestinal microbiota in ruminants, which are primarily involved in nutrient degradation, energy conversion, membrane transport, growth, and reproduction [9,38].

Notwithstanding this overall similarity, functional clustering and profile-based analyses indicated that predicted functional capacities varied across gastrointestinal segments in both species. Pathways such as glycolysis/gluconeogenesis, methane metabolism, pyruvate metabolism, ABC transporters, and two-component systems exhibited segment-associated variation in relative abundance, suggesting that metabolic configurations and regulatory processes differ along the gastrointestinal axis. This trend agrees with previous findings that different gastrointestinal segments in ruminants exhibit clear functional partitioning due to differences in substrate type, local microenvironment, and nutrient transport mode [22,39,40].

It should be emphasized that the present results are better suited to interpreting functional differences among segments in terms of overall shifts in functional profile rather than to overinterpretation of single pathways. On the one hand, although microbial composition differs among gastrointestinal segments, strong functional redundancy may allow different taxa to perform similar metabolic tasks, such that changes in individual pathways are not necessarily prominent [41,42]. PICRUSt2-based predictions are derived from 16S rRNA gene profiles and therefore provide indirect estimates of functional potential rather than direct measurements of gene expression or metabolic activity [43].

Therefore, the functional prediction results in this study indicate that potential functions of the microbiota vary among gastrointestinal segments in sika deer and reindeer at the level of the overall functional spectrum, but that these changes are reflected mainly in differences in functional combinations and relative contribution patterns rather than in significant increases or decreases in individual metabolic pathways. Future work integrating metagenomics, metabolomics, volatile fatty acid profiles, and methane emission indices may provide a more direct assessment of the biological significance of these predicted functional differences and help resolve the relationship between microbial composition and host metabolic outcomes [44,45].

A limitation of this study is the absence of reindeer ileal samples, which prevented direct interspecific comparison of the ileal microbiota. Therefore, the ileum was excluded from species-level comparisons and was only included in the within-species analysis of sika deer. Future studies should include matched ileal samples from both species to obtain a more complete view of microbial biogeography along the cervid gastrointestinal tract.

## 5. Conclusions

Based on 16S rRNA high-throughput sequencing, this study demonstrated distinct patterns in microbial community composition, community stratification, and predicted microbiota functions across different gastrointestinal segments in sika deer and reindeer. At the taxonomic level, methanogen-associated taxa were more prominent in the gastrointestinal tract of reindeer, whereas sika deer exhibited comparatively higher abundance of Bacteroidota-related taxa and fiber-degrading taxa. Meanwhile, both species exhibited obvious segmental differentiation among the foregut, small intestine, and hindgut, confirming that gastrointestinal location is an important factor shaping microbial structure. Differences in predicted functional capacity were expressed primarily through shifts in the overall functional profile and relative contributions of functional categories rather than through consistent changes in individual metabolic pathways. Overall, the microbiota of different gastrointestinal segments in sika deer and reindeer displayed clear species-specific and segment-specific patterns.

## Figures and Tables

**Table 1:** Basic information on the experimental animals and sampling design.

Species	No. of Animals	Sex	Age	Health Status	Sampling Sites	No. of Samples
Reindeer	3	Male	5	Healthy	Rumen, reticulum, omasum, abomasum, duodenum, jejunum, cecum, colon, rectum	27
Sika deer	3	Male	5	Healthy	Rumen, reticulum, omasum, abomasum, duodenum, jejunum, ileum, cecum, colon, rectum	30

**Table 2:** Summary of sequencing data.

Sample ID	Species	Sampling Site	Effective Sequences	Total Bases (bp)	Mean Length (bp)
X-LW1	Reindeer	Rumen	33,399	13,614,880	407.64
X-LW2	Reindeer	Rumen	35,035	14,363,456	409.97
X-LW3	Reindeer	Rumen	35,620	14,530,815	407.94
X-WW1	Reindeer	Reticulum	38,908	15,975,663	410.60
X-WW2	Reindeer	Reticulum	37,694	15,368,799	407.73
X-WW3	Reindeer	Reticulum	35,970	14,651,559	407.33
X-BW1	Reindeer	Omasum	34,251	14,097,498	411.59
X-BW2	Reindeer	Omasum	38,333	15,906,583	414.96
X-BW3	Reindeer	Omasum	39,030	16,102,047	412.56
X-ZW1	Reindeer	Abomasum	35,600	14,590,952	409.86
X-ZW2	Reindeer	Abomasum	33,244	13,610,197	409.40
X-ZW3	Reindeer	Abomasum	38,977	16,089,145	412.79
X-SEZC1	Reindeer	Duodenum	38,103	15,181,482	398.43
X-SEZC2	Reindeer	Duodenum	33,616	13,635,900	405.64
X-SEZC3	Reindeer	Duodenum	38,048	15,296,700	402.04
X-KC1	Reindeer	Jejunum	38,284	15,247,721	398.28
X-KC2	Reindeer	Jejunum	36,636	14,630,083	399.34
X-KC3	Reindeer	Jejunum	35,197	14,234,787	404.43
X-MC1	Reindeer	Cecum	38,518	15,614,134	405.37
X-MC2	Reindeer	Cecum	37,888	15,128,192	399.29
X-MC3	Reindeer	Cecum	34,535	13,971,285	404.55
X-JC1	Reindeer	Colon	37,966	15,234,603	401.27
X-JC2	Reindeer	Colon	36,770	14,544,951	395.57
X-JC3	Reindeer	Colon	34,793	14,136,194	406.29
X-ZC1	Reindeer	Rectum	37,257	15,139,462	406.35
X-ZC2	Reindeer	Rectum	35,894	14,438,375	402.25
X-ZC3	Reindeer	Rectum	37,892	15,472,981	408.34
M-LW1	Sika deer	Rumen	87,561	35,826,075	409.16
M-LW2	Sika deer	Rumen	88,502	36,038,105	407.20
M-LW3	Sika deer	Rumen	86,048	35,329,520	410.58
M-WW1	Sika deer	Reticulum	96,381	39,429,970	409.11
M-WW2	Sika deer	Reticulum	37,161	15,492,458	416.90
M-WW3	Sika deer	Reticulum	81,638	33,671,172	412.44
M-BW1	Sika deer	Omasum	85,231	35,413,658	415.50
M-BW2	Sika deer	Omasum	92,744	38,879,096	419.21
M-BW3	Sika deer	Omasum	37,114	15,512,033	417.96
M-ZW1	Sika deer	Abomasum	90,507	37,763,554	417.24
M-ZW2	Sika deer	Abomasum	38,790	16,052,831	413.84
M-ZW3	Sika deer	Abomasum	86,048	35,329,520	410.58
M-SEZC1	Sika deer	Duodenum	85,722	34,831,078	406.33
M-SEZC2	Sika deer	Duodenum	38,468	15,889,150	413.05
M-SEZC3	Sika deer	Duodenum	93,702	38,491,825	410.79
M-KC1	Sika deer	Jejunum	87,378	35,257,470	403.51
M-KC2	Sika deer	Jejunum	91,065	36,823,621	404.37
M-KC3	Sika deer	Jejunum	34,455	14,024,719	407.04
M-HC1	Sika deer	Ileum	92,614	38,327,783	413.84
M-HC2	Sika deer	Ileum	86,044	35,334,806	410.66
M-HC3	Sika deer	Ileum	35,973	14,801,493	411.46
M-MC1	Sika deer	Cecum	86,574	35,281,800	407.53
M-MC2	Sika deer	Cecum	37,038	15,040,810	406.09
M-MC3	Sika deer	Cecum	85,747	34,853,695	406.47
M-ZC1	Sika deer	Rectum	87,963	36,029,799	409.60
M-ZC2	Sika deer	Rectum	37,239	15,356,960	412.39
M-ZC3	Sika deer	Rectum	89,729	37,271,037	415.37

**Table 3:** Between-group differences in beta diversity of microbiota across gastrointestinal segments.

Species	Method	Distance Metric	Statistic	*p*-Value
Sika deer	ANOSIM	Bray–Curtis	R = 0.4084	0.001
Sika deer	PERMANOVA (Adonis)	Bray–Curtis	R^2^ = 0.4311, F = 1.684	0.005
Reindeer	ANOSIM	Bray–Curtis	R = 0.5766	0.001
Reindeer	PERMANOVA (Adonis)	Bray–Curtis	R^2^ = 0.62682, F = 3.7793	0.001

**Table 4:** Relative abundance of major functional categories.

KEGG Level 2 Functional Category	Reindeer (%)	Sika Deer (%)	KEGG Level 1
Carbohydrate metabolism	14.84	16.17	Metabolism
Amino acid metabolism	13.85	13.85	Metabolism
Energy metabolism	11.71	9.31	Metabolism
Translation	8.81	7.51	Genetic Information Processing
Nucleotide metabolism	8.31	7.78	Metabolism
Metabolism of cofactors and vitamins	7.22	7.06	Metabolism
Membrane transport	5.51	5.87	Environmental Information Processing
Replication and repair	5.40	6.12	Genetic Information Processing
Lipid metabolism	2.78	3.43	Metabolism
Signal transduction	2.28	2.54	Environmental Information Processing
Metabolism of other amino acids	2.35	2.46	Metabolism
Glycan biosynthesis and metabolism	2.07	2.34	Metabolism

**Table 5:** Between-group differences in overall functional profiles.

Species	Method	Distance Metric	Statistic	*p* Value
Sika deer	ANOSIM	Bray–Curtis	R = 0.2892	0.001
Sika deer	PERMANOVA (Adonis)	Bray–Curtis	R^2^ = 0.49943, F = 2.2171	0.004
Reindeer	ANOSIM	Bray–Curtis	R = 0.1433	0.075
Reindeer	PERMANOVA (Adonis)	Bray–Curtis	R^2^ = 0.46203, F = 1.9324	0.040

## Data Availability

The datasets produced and/or analyzed during the current study are available from the corresponding author upon reasonable request.

## Supplementary Materials

The following supporting information can be downloaded at: https://www.mdpi.com/article/10.3390/ani16101476/s1, Table S1: Alpha diversity indices of microbiota across gastrointestinal segments in reindeer and sika deer; Table S2: Student’s t-test results for alpha diversity indices between reindeer and sika deer in shared gastrointestinal segments; Table S3: LEfSe-identified differentially abundant taxa among gastrointestinal segments in sika deer and reindeer.

## References

[1] Urga, Wang X., Wei H., Zhao G. (2025). Mechanisms and applications of gastrointestinal microbiota-metabolite interactions in ruminants: A review. Microorganisms.

[2] Chen X., Yan F., Liu T., Zhang Y., Li X., Wang M., Zhang C., Xu X., Deng L., Yao J. (2022). Ruminal microbiota determines the high-fiber utilization of ruminants: Evidence from the ruminal microbiota transplant. Microbiol. Spectr..

[3] Xu Q., Qiao Q., Gao Y., Hou J., Hu M., Du Y., Zhao K., Li X. (2021). Gut microbiota and their role in health and metabolic disease of dairy cow. Front. Nutr..

[4] Silva É.B.R.d., Silva J.A.R.d., Silva W.C.d., Belo T.S., Sousa C.E.L., Santos M.R.P.d., Neves K.A.L., Rodrigues T.C.G.d.C., Camargo-Júnior R.N.C., Lourenço-Júnior J.d.B. (2024). A review of the rumen microbiota and the different molecular techniques used to identify microorganisms found in the rumen fluid of ruminants. Animals.

[5] Salgado-Flores A., Hagen L.H., Ishaq S.L., Zamanzadeh M., Wright A.-D.G., Pope P.B., Sundset M.A. (2016). Rumen and cecum microbiomes in reindeer (*Rangifer tarandus tarandus*) are changed in response to a lichen diet and may affect enteric methane emissions. PLoS ONE.

[6] Eto M., Yahara T., Kuroiwa A., Shioya K., Flores G.E., Hamamura N. (2022). Dynamics of rumen microbiome in sika deer (*Cervus nippon yakushimae*) from unique subtropical ecosystem in Yakushima Island, Japan. Sci. Rep..

[7] Wang Y., Shi M., Wu J., Han X., Li M., Wu Y., Jiang Y., Zhang H., Liu S., Hu D. (2025). Variations in intestinal microbiota among three species in the Cervidae family under the same feeding conditions. Vet. Sci..

[8] Li Z., Wright A.-D.G., Liu H., Fan Z., Yang F., Zhang Z., Li G. (2015). Response of the rumen microbiota of sika deer (*Cervus nippon*) fed different concentrations of tannin rich plants. PLoS ONE.

[9] Hu X., Wei Y., Zhang T., Wang X., Xu Y., Zhang W., Zheng Y. (2022). Gastrointestinal biogeography of luminal microbiota and short-chain fatty acids in sika deer (*Cervus nippon*). Appl. Environ. Microbiol..

[10] Tang L., Wen X., Zhang R., Xing X. (2022). Current situation and utilization of velvet deer germplasm resources in China. Animals.

[11] Ba H., Jia B., Wang G., Yang Y., Kedem G., Li C. (2017). Genome-wide SNP discovery and analysis of genetic diversity in farmed sika deer (*Cervus nippon*) in northeast China using double-digest restriction site-associated DNA sequencing. G3 Genes|Genomes|Genet.

[12] Loginov V.G., Ignatyeva M.N., Naumov I.V. (2022). Reindeer husbandry as a basic sector of the traditional economy of indigenous ethnic groups: Present and future. Reg. Sci. Policy Pract..

[13] Kim J.H., Hong S.W., Park B.Y., Yoo J.G., Oh M.H. (2019). Characterisation of the bacterial community in the gastrointestinal tracts of elk (*Cervus canadensis*). Antonie Van. Leeuwenhoek.

[14] Maritan E., Quagliariello A., Frago E., Patarnello T., Martino M.E. (2024). The role of animal hosts in shaping gut microbiome variation. Philos. Trans. R. Soc. B Biol. Sci..

[15] Martínez-Álvaro M., Auffret M.D., Duthie C.A., Dewhurst R.J., Cleveland M.A., Watson M., Roehe R. (2022). Bovine host genome acts on rumen microbiome function linked to methane emissions. Commun. Biol..

[16] Pacheco-Torres I., Hernández-Sánchez D., García-De la Peña C., Tarango-Arámbula L.A., Crosby-Galván M.M., Sánchez-Santillán P. (2023). Analysis of the intestinal and faecal bacterial microbiota of the Cervidae family using 16s next-generation sequencing: A review. Microorganisms.

[17] He H., Fang C., Liu L., Li M., Liu W. (2024). Environmental driving of adaptation mechanism on rumen microorganisms of sheep based on metagenomics and metabolomics data analysis. Int. J. Mol. Sci..

[18] Li Z., Wang X., Zhang T., Si H., Xu C., Wright A.G., Li G. (2019). Heterogeneous development of methanogens and the correlation with bacteria in the rumen and cecum of sika deer (*Cervus nippon*) during early life suggest different ecology relevance. BMC Microbiol..

[19] Betancur-Murillo C.L., Aguilar-Marín S.B., Jovel J. (2022). Prevotella: A key player in ruminal metabolism. Microorganisms.

[20] Li B., Jia G., Wen D., Zhao X., Zhang J., Xu Q., Zhao X., Jiang N., Liu Z., Wang Y. (2022). Rumen microbiota of indigenous and introduced ruminants and their adaptation to the Qinghai-Tibetan plateau. Front. Microbiol..

[21] Tardiolo G., La Fauci D., Riggio V., Daghio M., Di Salvo E., Zumbo A., Sutera A.M. (2025). Gut microbiota of ruminants and monogastric livestock: An overview. Animals.

[22] Liu Y., Shu Y., Huang Y., Tan J., Wang F., Tang L., Fang T., Yuan S., Wang L. (2024). Microbial biogeography along the gastrointestinal tract of a wild Chinese muntjac (*Muntiacus reevesi*). Microorganisms.

[23] Perez H.G., Stevenson C.K., Lourenco J.M., Callaway T.R. (2024). Understanding rumen microbiology: An overview. Encyclopedia.

[24] Dawson P.A., Karpen S.J. (2015). Intestinal transport and metabolism of bile acids. J. Lipid Res..

[25] Li Z., Wang X., Zhang T., Si H., Nan W., Xu C., Guan L., Wright A.-D.G., Li G. (2018). The development of microbiota and metabolome in small intestine of sika deer (*Cervus nippon*) from birth to weaning. Front. Microbiol..

[26] Song Y., Malmuthuge N., Steele M.A., Guan L.L. (2018). Shift of hindgut microbiota and microbial short chain fatty acids profiles in dairy calves from birth to pre-weaning. FEMS Microbiol. Ecol..

[27] Han X., Lei X., Yang X., Shen J., Zheng L., Jin C., Cao Y., Yao J. (2021). A metagenomic insight into the hindgut microbiota and their metabolites for dairy goats fed different rumen degradable starch. Front. Microbiol..

[28] Zhang Y., Cheng J., Lin C., Li F., Zhang X., Li C., Zhang D., Yang X., Xu D., Zhao Y. (2025). Spatial heterogeneity determines the gastrointestinal microbiome signatures and ecological processes that govern bacterial community assembly in sheep. Microbiol. Spectr..

[29] Cao Z., Wang D., Hu X., He J., Liu Y., Liu W., Zhan J., Bao Z., Guo C., Xu Y. (2024). Comparison and association of winter diets and gut microbiota using trnL and 16S rRNA gene sequencing for three herbivores in Taohongling, China. Glob. Ecol. Conserv..

[30] Xia Y. (2023). Statistical normalization methods in microbiome data with application to microbiome cancer research. Gut Microbes.

[31] Bokulich N.A., Ziemski M., Robeson M.S., Kaehler B.D. (2020). Measuring the microbiome: Best practices for developing and benchmarking microbiomics methods. Comput. Struct. Biotechnol. J..

[32] Fu R., Han L., Li Q., Li Z., Dai Y., Leng J. (2025). Studies on the concerted interaction of microbes in the gastrointestinal tract of ruminants on lignocellulose and its degradation mechanism. Front. Microbiol..

[33] Weimer P.J. (2022). Degradation of cellulose and hemicellulose by ruminal microorganisms. Microorganisms.

[34] Singh V., Lee G., Son H., Koh H., Kim E.S., Unno T., Shin J.-H. (2022). Butyrate producers, “the sentinel of gut”: Their intestinal significance with and beyond butyrate, and prospective use as microbial therapeutics. Front. Microbiol..

[35] Nie K., Ma K., Luo W., Shen Z., Yang Z., Xiao M., Tong T., Yang Y., Wang X. (2021). Roseburia intestinalis: A beneficial gut organism from the discoveries in genus and species. Front. Cell. Infect. Microbiol..

[36] Grondin J.M., Tamura K., Déjean G., Abbott D.W., Brumer H. (2017). Polysaccharide utilization loci: Fueling microbial communities. J. Bacteriol..

[37] Kaplan H., Hutkins R.W. (2000). Fermentation of fructooligosaccharides by lactic acid bacteria and bifidobacteria. Appl. Environ. Microbiol..

[38] Douglas G.M., Maffei V.J., Zaneveld J.R., Yurgel S.N., Brown J.R., Taylor C.M., Huttenhower C., Langille M.G.I. (2020). Picrust2 for prediction of metagenome functions. Nat. Biotechnol..

[39] Cao Y., Feng T., Wu Y., Xu Y., Du L., Wang T., Luo Y., Wang Y., Li Z., Xuan Z. (2023). The multi-kingdom microbiome of the goat gastrointestinal tract. Microbiome.

[40] Wang L., Jin L., Xue B., Wang Z., Peng Q. (2019). Characterizing the bacterial community across the gastrointestinal tract of goats: Composition and potential function. Microbiologyopen.

[41] Sun S., Jones R.B., Fodor A.A. (2020). Inference-based accuracy of metagenome prediction tools varies across sample types and functional categories. Microbiome.

[42] Gregor R., Probst M., Eyal S., Aksenov A., Sasson G., Horovitz I., Dorrestein P.C., Meijler M.M., Mizrahi I. (2022). Mammalian gut metabolomes mirror microbiome composition and host phylogeny. ISME J..

[43] Kers J.G., Saccenti E. (2022). The power of microbiome studies: Some considerations on which alpha and beta metrics to use and how to report results. Front. Microbiol..

[44] Yan X., Si H., Zhu Y., Li S., Han Y., Liu H., Du R., Pope P.B., Qiu Q., Li Z. (2022). Integrated multi-omics of the gastrointestinal microbiome and ruminant host reveals metabolic adaptation underlying early life development. Microbiome.

[45] Zhu Y., Chai Y., Chen S., Qian W., Si H., Li Z. (2026). Distinct rumen microbial features and host metabolic responses in three cervid species. Animals.

