# Supplementary material for: Comparative Analysis of Gastrointestinal Microbiota Along the Digestive Tract in Sika Deer and Reindeer and Prediction of Their Potential Function
Source: Animals (Basel). 2026 May 11;16(10):1476. doi: 10.3390/ani16101476 (PMC13203257; doi:10.3390/ani16101476)
Supplement: Supplementary file 1 [file animals-16-01476-s001.zip › Supplementary Materials Table S3.pdf]

Supplementary Materials

Title: Comparative Analysis of Gastrointestinal Microbiota Along the Digestive Tract in Sika Deer and Reindeer and Prediction of Their Potential Functions

Authors: Xinyu Peng, Huansheng Han, Ruihong Hu, Fanzhi Kong, and Yuhan Lu

Corresponding Author: Huansheng Han

Supplementary Table S3. LEfSe-identified differentially abundant taxa among gastrointestinal segments in sika deer and reindeer.

Note: Due to the large size of the dataset, this table is provided as a separate supplementary file.

Abbreviations: X, reindeer; M, sika deer; LW, rumen; WW, reticulum; BW, omasum; ZW, abomasum; SEZC, duodenum; KC, jejunum; HC, ileum; MC, cecum; JC, colon; ZC, rectum; LEfSe, linear discriminant analysis effect size; LDA, linear discriminant analysis.

Taxa with LDA score > 2.0 and P < 0.05 were considered differentially abundant.

Table S3. LEfSe-identified differentially abundant taxa among gastrointestinal segments in sika deer and reindeer.

Species	Taxon	Taxonomic level	Enriched group	Segment	Original LEfSe abundance score	LDA score (log10)	P value	Full taxonomy
Sika deer	Prevotellaceae	Family	M-BW	Omasum	6.277	5.815	0.0256	Bacteria > Bacteroidota > Bacteroidia > Bacteroidales > Prevotellaceae
Sika deer	Saccharimonadales	Order	M-LW	Rumen	7.444	7.082	0.0340	Bacteria > Patescibacteria > Saccharimonadia > Saccharimonadales
Sika deer	Patescibacteria	Phylum	M-LW	Rumen	7.444	7.080	0.0340	Bacteria > Patescibacteria
Sika deer	Saccharimonadaceae	Family	M-LW	Rumen	7.444	7.050	0.0340	Bacteria > Patescibacteria > Saccharimonadia > Saccharimonadales > Saccharimonadaceae
Sika deer	Saccharimonadia	Class	M-LW	Rumen	7.444	7.038	0.0340	Bacteria > Patescibacteria > Saccharimonadia
Sika deer	Lactobacillales	Order	M-LW	Rumen	5.245	4.994	0.0444	Bacteria > Bacillota > Bacilli > Lactobacillales
Sika deer	*Alloprevotella*	Genus	M-SEZC	Duodenum	5.733	5.340	0.0428	Bacteria > Bacteroidota > Bacteroidia > Bacteroidales > Prevotellaceae > Alloprevotella
Sika deer	Williamwhitmaniaceae	Family	M-WW	Reticulum	6.159	5.874	0.0295	Bacteria > Bacteroidota > Bacteroidia > Bacteroidales > Williamwhitmaniaceae
Sika deer	Bacilli	Class	M-WW	Reticulum	5.238	5.230	0.0191	Bacteria > Bacillota > Bacilli
Sika deer	Streptococcaceae	Family	M-WW	Reticulum	4.926	5.048	0.0371	Bacteria > Bacillota > Bacilli > Lactobacillales > Streptococcaceae
Sika deer	*Parabacteroides*	Genus	M-ZW	Abomasum	6.043	5.714	0.0382	Bacteria > Bacteroidota > Bacteroidia > Bacteroidales > Tannerellaceae > Parabacteroides
Sika deer	Lachnospiraceae UCG-008	Genus	M-ZW	Abomasum	5.455	5.387	0.0483	Bacteria > Bacillota > Clostridia > Lachnospirales > Lachnospiraceae > Lachnospiraceae UCG 008
Reindeer	Bacteroidota	Phylum	X-BW	Omasum	6.484	6.068	0.0073	Bacteria > Bacteroidota
Reindeer	Prevotellaceae	Family	X-BW	Omasum	6.223	5.849	0.0065	Bacteria > Bacteroidota > Bacteroidia > Bacteroidales > Prevotellaceae
Reindeer	Bacteroidia	Class	X-BW	Omasum	5.702	5.303	0.0066	Bacteria > Bacteroidota > Bacteroidia
Reindeer	Bacteroidales	Order	X-BW	Omasum	5.082	4.690	0.0085	Bacteria > Bacteroidota > Bacteroidia > Bacteroidales
Reindeer	*Acetobacteroides*	Genus	X-BW	Omasum	4.862	4.484	0.0063	Bacteria > Bacteroidota > Bacteroidia > Bacteroidales > Williamwhitmaniaceae > Acetobacteroides
Reindeer	Barnesiellaceae	Family	X-BW	Omasum	4.527	4.114	0.0250	Bacteria > Bacteroidota > Bacteroidia > Bacteroidales > Barnesiellaceae
Reindeer	*Roseburia*	Genus	X-BW	Omasum	4.100	3.680	0.0145	Bacteria > Bacillota > Clostridia > Lachnospirales > Lachnospiraceae > Roseburia
Reindeer	Prevotellaceae NK3B31 group	Genus	X-BW	Omasum	3.972	3.608	0.0054	Bacteria > Bacteroidota > Bacteroidia > Bacteroidales > Prevotellaceae > Prevotellaceae NK3B31 group
Reindeer	*Prevotella ruminicola*	Species	X-BW	Omasum	3.952	3.476	0.0111	Bacteria > Bacteroidota > Bacteroidia > Bacteroidales > Prevotellaceae > Xylanibacter > Prevotella ruminicola
Reindeer	Veillonellaceae UCG-001	Genus	X-BW	Omasum	3.804	3.431	0.0116	Bacteria > Bacillota > Negativicutes > Veillonellales Selenomonadales > Selenomonadaceae > Veillonellaceae UCG 001
Reindeer	*Butyrivibrio*	Genus	X-BW	Omasum	3.722	3.352	0.0035	Bacteria > Bacillota > Clostridia > Lachnospirales > Lachnospiraceae > Butyrivibrio
Reindeer	Leucotrichaceae	Family	X-BW	Omasum	3.710	3.326	0.0039	Bacteria > Pseudomonadota > Gammaproteobacteria > Beggiatoales > Leucotrichaceae
Reindeer	*Thiothrix*	Genus	X-BW	Omasum	3.710	3.302	0.0039	Bacteria > Pseudomonadota > Gammaproteobacteria > Beggiatoales > Leucotrichaceae > Thiothrix
Reindeer	Beggiatoales	Order	X-BW	Omasum	3.710	3.287	0.0039	Bacteria > Pseudomonadota > Gammaproteobacteria > Beggiatoales
Reindeer	Prevotellaceae Ga6A1 group	Genus	X-BW	Omasum	3.366	3.017	0.0410	Bacteria > Bacteroidota > Bacteroidia > Bacteroidales > Prevotellaceae > Prevotellaceae Ga6A1 group
Reindeer	Eubacterium ruminantium group	Genus	X-BW	Omasum	3.282	2.968	0.0063	Bacteria > Bacillota > Clostridia > Lachnospirales > Lachnospiraceae > Eubacterium ruminantium group
Reindeer	V9D2013 group	Genus	X-BW	Omasum	3.265	2.902	0.0420	Bacteria > Bacillota > Clostridia > Oscillospirales > Oscillospiraceae > V9D2013 group
Reindeer	Bacteroidaceae	Family	X-JC	Colon	4.458	4.151	0.0053	Bacteria > Bacteroidota > Bacteroidia > Bacteroidales > Bacteroidaceae
Reindeer	*Ruminiclostridium*	Genus	X-JC	Colon	3.926	3.621	0.0082	Bacteria > Bacillota > Clostridia > Oscillospirales > Oscillospiraceae > Ruminiclostridium
Reindeer	*Oscillibacter*	Genus	X-JC	Colon	3.861	3.558	0.0087	Bacteria > Bacillota > Clostridia > Oscillospirales > Oscillospiraceae > Oscillibacter
Reindeer	Dysgonomonadaceae	Family	X-JC	Colon	3.677	3.389	0.0197	Bacteria > Bacteroidota > Bacteroidia > Bacteroidales > Dysgonomonadaceae
Reindeer	*Dysgonomonas*	Genus	X-JC	Colon	3.677	3.350	0.0197	Bacteria > Bacteroidota > Bacteroidia > Bacteroidales > Dysgonomonadaceae > Dysgonomonas
Reindeer	*Kineothrix*	Genus	X-JC	Colon	3.642	3.338	0.0167	Bacteria > Bacillota > Clostridia > Lachnospirales > Lachnospiraceae > Kineothrix
Reindeer	DTU089	Genus	X-JC	Colon	2.939	2.979	0.0193	Bacteria > Bacillota > Clostridia > Oscillospirales > Ruminococcaceae > DTU089
Reindeer	Lachnospirales	Order	X-LW	Rumen	6.031	5.680	0.0427	Bacteria > Bacillota > Clostridia > Lachnospirales
Reindeer	Peptostreptococcales Tissierellales	Order	X-LW	Rumen	5.345	5.013	0.0487	Bacteria > Bacillota > Clostridia > Peptostreptococcales Tissierellales
Reindeer	Christensenellaceae R-7 group	Genus	X-LW	Rumen	4.795	4.462	0.0465	Bacteria > Bacillota > Clostridia > Christensenellales > Christensenellaceae > Christensenellaceae R 7 group
Reindeer	*Butyrivibrio fibrisolvens*	Species	X-LW	Rumen	4.855	4.446	0.0085	Bacteria > Bacillota > Clostridia > Lachnospirales > Lachnospiraceae > Pseudobutyrivibrio > Butyrivibrio fibrisolvens
Reindeer	Family XIII AD3011 group	Genus	X-LW	Rumen	4.671	4.372	0.0036	Bacteria > Bacillota > Clostridia > Peptostreptococcales Tissierellales > Anaerovoracaceae > Family XIII AD3011 group
Reindeer	Lachnospiraceae NC2004 group	Genus	X-LW	Rumen	4.643	4.350	0.0053	Bacteria > Bacillota > Clostridia > Lachnospirales > Lachnospiraceae > Lachnospiraceae NC2004 group
Reindeer	Peptococcaceae	Family	X-LW	Rumen	4.598	4.305	0.0083	Bacteria > Bacillota > Clostridia > Peptococcales > Peptococcaceae
Reindeer	Sphingobacteriales	Order	X-LW	Rumen	4.577	4.287	0.0077	Bacteria > Bacteroidota > Bacteroidia > Sphingobacteriales
Reindeer	*Pseudobutyrivibrio*	Genus	X-LW	Rumen	4.682	4.277	0.0119	Bacteria > Bacillota > Clostridia > Lachnospirales > Lachnospiraceae > Pseudobutyrivibrio
Reindeer	Peptococcales	Order	X-LW	Rumen	4.598	4.263	0.0083	Bacteria > Bacillota > Clostridia > Peptococcales
Reindeer	*Dehalobacterium formicoaceticum*	Species	X-LW	Rumen	4.571	4.260	0.0063	Bacteria > Bacillota > Clostridia > Peptococcales > Peptococcaceae > Dehalobacterium > Dehalobacterium formicoaceticum
Reindeer	Lentimicrobiaceae	Family	X-LW	Rumen	4.569	4.260	0.0074	Bacteria > Bacteroidota > Bacteroidia > Sphingobacteriales > Lentimicrobiaceae
Reindeer	Lachnospiraceae UCG-006	Genus	X-LW	Rumen	4.568	4.251	0.0053	Bacteria > Bacillota > Clostridia > Lachnospirales > Lachnospiraceae > Lachnospiraceae UCG 006
Reindeer	Erysipelotrichales	Order	X-LW	Rumen	4.600	4.249	0.0146	Bacteria > Bacillota > Bacilli > Erysipelotrichales
Reindeer	Lachnospiraceae XPB1014 group	Genus	X-LW	Rumen	4.550	4.230	0.0055	Bacteria > Bacillota > Clostridia > Lachnospirales > Lachnospiraceae > Lachnospiraceae XPB1014 group
Reindeer	Lachnospiraceae NK3A20 group	Genus	X-LW	Rumen	4.552	4.208	0.0031	Bacteria > Bacillota > Clostridia > Lachnospirales > Lachnospiraceae > Lachnospiraceae NK3A20 group
Reindeer	Rikenellaceae RC9 gut group	Genus	X-LW	Rumen	4.480	4.161	0.0071	Bacteria > Bacteroidota > Bacteroidia > Bacteroidales > Rikenellaceae > Rikenellaceae RC9 gut group
Reindeer	*Shuttleworthia*	Genus	X-LW	Rumen	4.323	4.018	0.0098	Bacteria > Bacillota > Clostridia > Lachnospirales > Lachnospiraceae > Shuttleworthia
Reindeer	Eubacterium oxidoreducens group	Genus	X-LW	Rumen	4.317	4.010	0.0195	Bacteria > Bacillota > Clostridia > Lachnospirales > Lachnospiraceae > Eubacterium oxidoreducens group
Reindeer	Desulfovibrionaceae	Family	X-LW	Rumen	4.322	4.008	0.0492	Bacteria > Thermodesulfobacteriota > Desulfovibrionia > Desulfovibrionales > Desulfovibrionaceae
Reindeer	Desulfovibrionia	Class	X-LW	Rumen	4.322	3.995	0.0492	Bacteria > Thermodesulfobacteriota > Desulfovibrionia
Reindeer	Lachnospiraceae NK4B4 group	Genus	X-LW	Rumen	4.331	3.994	0.0230	Bacteria > Bacillota > Clostridia > Lachnospirales > Lachnospiraceae > Lachnospiraceae NK4B4 group
Reindeer	*Syntrophococcus*	Genus	X-LW	Rumen	4.309	3.988	0.0051	Bacteria > Bacillota > Clostridia > Lachnospirales > Lachnospiraceae > Syntrophococcus
Reindeer	Desulfovibrionales	Order	X-LW	Rumen	4.322	3.985	0.0492	Bacteria > Thermodesulfobacteriota > Desulfovibrionia > Desulfovibrionales

Species	Taxon	Taxonomic level	Enriched group	Segment	Original LEfSe abundance score	LDA score (log10)	P value	Full taxonomy
Reindeer	*Lentimicrobium*	Genus	X-LW	Rumen	4.230	3.937	0.0086	Bacteria > Bacteroidota > Bacteroidia > Sphingobacteriales > Lentimicrobiaceae > Lentimicrobium
Reindeer	Defluviitaleaceae	Family	X-LW	Rumen	4.164	3.864	0.0046	Bacteria > Bacillota > Clostridia > Lachnospirales > Defluviitaleaceae
Reindeer	Atopobiaceae	Family	X-LW	Rumen	4.158	3.862	0.0421	Bacteria > Actinomycetota > Coriobacteriia > Coriobacteriales > Atopobiaceae
Reindeer	*Saccharofermentans*	Genus	X-LW	Rumen	4.171	3.847	0.0027	Bacteria > Bacillota > Clostridia > Oscillospirales > Oscillospiraceae > Saccharofermentans
Reindeer	Defluviitaleaceae UCG-011	Genus	X-LW	Rumen	4.164	3.842	0.0046	Bacteria > Bacillota > Clostridia > Lachnospirales > Defluviitaleaceae > Defluviitaleaceae UCG 011
Reindeer	Eubacterium siraeum group	Genus	X-LW	Rumen	4.106	3.807	0.0059	Bacteria > Bacillota > Clostridia > Oscillospirales > Ruminococcaceae > Eubacterium siraeum group
Reindeer	*Oribacterium*	Genus	X-LW	Rumen	4.063	3.763	0.0029	Bacteria > Bacillota > Clostridia > Lachnospirales > Lachnospiraceae > Oribacterium
Reindeer	*Barnesiella*	Genus	X-LW	Rumen	4.082	3.734	0.0141	Bacteria > Bacteroidota > Bacteroidia > Bacteroidales > Barnesiellaceae > Barnesiella
Reindeer	Lachnospiraceae FCS020 group	Genus	X-LW	Rumen	4.030	3.680	0.0065	Bacteria > Bacillota > Clostridia > Lachnospirales > Lachnospiraceae > Lachnospiraceae FCS020 group
Reindeer	Enterococcaceae	Family	X-LW	Rumen	3.954	3.650	0.0186	Bacteria > Bacillota > Bacilli > Lactobacillales > Enterococcaceae
Reindeer	*Enterococcus mundtii*	Species	X-LW	Rumen	3.943	3.632	0.0171	Bacteria > Bacillota > Bacilli > Lactobacillales > Enterococcaceae > Enterococcus > Enterococcus mundtii
Reindeer	Blvii28 wastewater sludge group	Genus	X-LW	Rumen	3.857	3.573	0.0153	Bacteria > Bacteroidota > Bacteroidia > Bacteroidales > Williamwhitmaniaceae > Blvii28 wastewater sludge group
Reindeer	*Olsenella*	Genus	X-LW	Rumen	3.885	3.569	0.0354	Bacteria > Actinomycetota > Coriobacteriia > Coriobacteriales > Atopobiaceae > Olsenella
Reindeer	*Pseudoprevotella*	Genus	X-LW	Rumen	3.826	3.549	0.0058	Bacteria > Bacteroidota > Bacteroidia > Bacteroidales > Prevotellaceae > Pseudoprevotella
Reindeer	Family XIII UCG-001	Genus	X-LW	Rumen	3.851	3.546	0.0118	Bacteria > Bacillota > Clostridia > Peptostreptococcales Tissierellales > Anaerovoracaceae > Family XIII UCG 001
Reindeer	Eubacterium brachy group	Genus	X-LW	Rumen	3.835	3.498	0.0364	Bacteria > Bacillota > Clostridia > Peptostreptococcales Tissierellales > Anaerovoracaceae > Eubacterium brachy group
Reindeer	Lachnospiraceae UCG-008	Genus	X-LW	Rumen	3.783	3.486	0.0103	Bacteria > Bacillota > Clostridia > Lachnospirales > Lachnospiraceae > Lachnospiraceae UCG 008
Reindeer	*Lacrimispora*	Genus	X-LW	Rumen	3.746	3.472	0.0118	Bacteria > Bacillota > Clostridia > Lachnospirales > Lachnospiraceae > Lacrimispora
Reindeer	*Moryella*	Genus	X-LW	Rumen	3.742	3.425	0.0056	Bacteria > Bacillota > Clostridia > Lachnospirales > Lachnospiraceae > Moryella
Reindeer	NK4A214 group	Genus	X-LW	Rumen	3.699	3.378	0.0111	Bacteria > Bacillota > Clostridia > Oscillospirales > Oscillospiraceae > NK4A214 group
Reindeer	*Mogibacterium*	Genus	X-LW	Rumen	3.674	3.369	0.0482	Bacteria > Bacillota > Clostridia > Peptostreptococcales Tissierellales > Anaerovoracaceae > Mogibacterium
Reindeer	Anaerovoracaceae	Family	X-LW	Rumen	3.691	3.367	0.0427	Bacteria > Bacillota > Clostridia > Peptostreptococcales Tissierellales > Anaerovoracaceae
Reindeer	probable genus 10	Genus	X-LW	Rumen	3.660	3.362	0.0046	Bacteria > Bacillota > Clostridia > Lachnospirales > Lachnospiraceae > probable genus 10
Reindeer	*Mediterraneibacter*	Genus	X-LW	Rumen	3.586	3.339	0.0104	Bacteria > Bacillota > Clostridia > Lachnospirales > Lachnospiraceae > Mediterraneibacter
Reindeer	Lachnospiraceae FE2018 group	Genus	X-LW	Rumen	2.755	3.339	0.0066	Bacteria > Bacillota > Clostridia > Lachnospirales > Lachnospiraceae > Lachnospiraceae FE2018 group
Reindeer	*Ruthenibacterium*	Genus	X-LW	Rumen	3.545	3.249	0.0101	Bacteria > Bacillota > Clostridia > Oscillospirales > Ruminococcaceae > Ruthenibacterium
Reindeer	Desulfobulbales	Order	X-LW	Rumen	3.498	3.243	0.0064	Bacteria > Thermodesulfobacteriota > Desulfobulbia > Desulfobulbales
Reindeer	*Desulfobulbus*	Genus	X-LW	Rumen	3.498	3.221	0.0064	Bacteria > Thermodesulfobacteriota > Desulfobulbia > Desulfobulbales > Desulfobulbaceae > Desulfobulbus
Reindeer	Desulfobulbia	Class	X-LW	Rumen	3.498	3.217	0.0064	Bacteria > Thermodesulfobacteriota > Desulfobulbia
Reindeer	*Lachnoclostridium*	Genus	X-LW	Rumen	3.468	3.210	0.0118	Bacteria > Bacillota > Clostridia > Lachnospirales > Lachnospiraceae > Lachnoclostridium
Reindeer	Desulfobulbaceae	Family	X-LW	Rumen	3.498	3.199	0.0064	Bacteria > Thermodesulfobacteriota > Desulfobulbia > Desulfobulbales > Desulfobulbaceae
Reindeer	*Acidiphilium*	Genus	X-LW	Rumen	3.470	3.166	0.0197	Bacteria > Pseudomonadota > Alphaproteobacteria > Acetobacterales > Acetobacteraceae > Acidiphilium
Reindeer	Erysipelotrichaceae	Family	X-LW	Rumen	3.361	3.128	0.0101	Bacteria > Bacillota > Bacilli > Erysipelotrichales > Erysipelotrichaceae
Reindeer	*Blautia*	Genus	X-LW	Rumen	3.391	3.100	0.0192	Bacteria > Bacillota > Clostridia > Lachnospirales > Lachnospiraceae > Blautia
Reindeer	*Olsenella profusa*	Species	X-LW	Rumen	3.381	3.086	0.0366	Bacteria > Actinomycetota > Coriobacteriia > Coriobacteriales > Atopobiaceae > Olsenella > Olsenella profusa
Reindeer	*Dehalobacterium*	Genus	X-LW	Rumen	3.381	3.072	0.0392	Bacteria > Bacillota > Clostridia > Peptococcales > Peptococcaceae > Dehalobacterium
Reindeer	Eubacterium saphenum group	Genus	X-LW	Rumen	3.334	3.068	0.0113	Bacteria > Bacillota > Clostridia > Peptostreptococcales Tissierellales > Anaerovoracaceae > Eubacterium saphenum group
Reindeer	*Muribaculum intestinale*	Species	X-LW	Rumen	3.247	2.999	0.0432	Bacteria > Bacteroidota > Bacteroidia > Bacteroidales > Muribaculaceae > Muribaculum > Muribaculum intestinale
Reindeer	Lachnospiraceae ND3007 group	Genus	X-LW	Rumen	3.213	2.994	0.0187	Bacteria > Bacillota > Clostridia > Lachnospirales > Lachnospiraceae > Lachnospiraceae ND3007 group
Reindeer	*Anaerorhabdus*	Genus	X-LW	Rumen	3.071	2.982	0.0334	Bacteria > Bacillota > Bacilli > Erysipelotrichales > Erysipelotrichaceae > Anaerorhabdus
Reindeer	Acholeplasmatales	Order	X-LW	Rumen	3.066	2.948	0.0339	Bacteria > Bacillota > Bacilli > Acholeplasmatales
Reindeer	Cyanobacteriia	Class	X-LW	Rumen	2.906	2.856	0.0110	Bacteria > Cyanobacteriota > Cyanobacteriia
Reindeer	*Anaerotruncus*	Genus	X-LW	Rumen	2.936	2.833	0.0394	Bacteria > Bacillota > Clostridia > Oscillospirales > Ruminococcaceae > Anaerotruncus
Reindeer	*Syntrophococcus sucromutans*	Species	X-LW	Rumen	3.020	2.786	0.0285	Bacteria > Bacillota > Clostridia > Lachnospirales > Lachnospiraceae > Syntrophococcus > Syntrophococcus sucromutans
Reindeer	Monoglobaceae	Family	X-MC	Cecum	4.685	4.359	0.0181	Bacteria > Bacillota > Clostridia > Monoglobales > Monoglobaceae
Reindeer	Monoglobales	Order	X-MC	Cecum	4.685	4.343	0.0181	Bacteria > Bacillota > Clostridia > Monoglobales
Reindeer	*Tyzzerella*	Genus	X-MC	Cecum	4.418	4.088	0.0082	Bacteria > Bacillota > Clostridia > Lachnospirales > Lachnospiraceae > Tyzzerella
Reindeer	Clostridiales	Order	X-MC	Cecum	4.307	3.969	0.0050	Bacteria > Bacillota > Clostridia > Clostridiales
Reindeer	Butyricicoccaceae	Family	X-MC	Cecum	4.278	3.956	0.0060	Bacteria > Bacillota > Clostridia > Oscillospirales > Butyricicoccaceae
Reindeer	Clostridiaceae	Family	X-MC	Cecum	4.306	3.927	0.0049	Bacteria > Bacillota > Clostridia > Clostridiales > Clostridiaceae
Reindeer	Lachnospiraceae NK4A136 group	Genus	X-MC	Cecum	4.191	3.868	0.0213	Bacteria > Bacillota > Clostridia > Lachnospirales > Lachnospiraceae > Lachnospiraceae NK4A136 group
Reindeer	*Flavonifractor*	Genus	X-MC	Cecum	4.091	3.760	0.0093	Bacteria > Bacillota > Clostridia > Oscillospirales > Oscillospiraceae > Flavonifractor
Reindeer	*Alistipes*	Genus	X-MC	Cecum	3.858	3.578	0.0269	Bacteria > Bacteroidota > Bacteroidia > Bacteroidales > Rikenellaceae > Alistipes
Reindeer	*Pygmaiobacter*	Genus	X-MC	Cecum	3.652	3.384	0.0340	Bacteria > Bacillota > Clostridia > Oscillospirales > Ruminococcaceae > Pygmaiobacter
Reindeer	*Terrisporobacter*	Genus	X-MC	Cecum	3.626	3.336	0.0187	Bacteria > Bacillota > Clostridia > Peptostreptococcales Tissierellales > Peptostreptococcaceae > Terrisporobacter
Reindeer	*Dorea*	Genus	X-MC	Cecum	3.510	3.271	0.0166	Bacteria > Bacillota > Clostridia > Lachnospirales > Lachnospiraceae > Dorea
Reindeer	*Acutalibacter*	Genus	X-MC	Cecum	3.448	3.268	0.0061	Bacteria > Bacillota > Clostridia > Oscillospirales > Ruminococcaceae > Acutalibacter
Reindeer	Verrucomicrobiia	Class	X-MC	Cecum	3.573	3.267	0.0309	Bacteria > Verrucomicrobiota > Verrucomicrobiia
Reindeer	*Coprococcus*	Genus	X-MC	Cecum	3.313	3.068	0.0307	Bacteria > Bacillota > Clostridia > Lachnospirales > Lachnospiraceae > Coprococcus
Reindeer	Fusobacteriota	Phylum	X-SEZC	Duodenum	3.826	3.529	0.0105	Bacteria > Fusobacteriota
Reindeer	*Fusobacterium*	Genus	X-SEZC	Duodenum	3.826	3.505	0.0079	Bacteria > Fusobacteriota > Fusobacteriia > Fusobacteriales > Fusobacteriaceae > Fusobacterium

Species	Taxon	Taxonomic level	Enriched group	Segment	Original LEfSe abundance score	LDA score (log10)	P value	Full taxonomy
Reindeer	*Fusobacterium necrophorum subsp funduliforme*	Species	X-SEZC	Duodenum	3.826	3.501	0.0079	Bacteria > Fusobacteriota > Fusobacteriia > Fusobacteriales > Fusobacteriaceae > Fusobacterium > Fusobacterium necrophorum subsp funduliforme
Reindeer	Fusobacteriales	Order	X-SEZC	Duodenum	3.826	3.494	0.0105	Bacteria > Fusobacteriota > Fusobacteriia > Fusobacteriales
Reindeer	Gastranaerophilaceae	Family	X-SEZC	Duodenum	3.877	3.494	0.0213	Bacteria > Cyanobacteriota > Vampirivibrionia > Gastranaerophilales > Gastranaerophilaceae
Reindeer	Fusobacteriaceae	Family	X-SEZC	Duodenum	3.826	3.493	0.0079	Bacteria > Fusobacteriota > Fusobacteriia > Fusobacteriales > Fusobacteriaceae
Reindeer	Fusobacteriia	Class	X-SEZC	Duodenum	3.826	3.491	0.0105	Bacteria > Fusobacteriota > Fusobacteriia
Reindeer	Gastranaerophilales	Order	X-SEZC	Duodenum	3.877	3.481	0.0213	Bacteria > Cyanobacteriota > Vampirivibrionia > Gastranaerophilales
Reindeer	*Cryobacterium*	Genus	X-SEZC	Duodenum	3.778	3.447	0.0314	Bacteria > Actinomycetota > Actinobacteria > Micrococcales > Microbacteriaceae > Cryobacterium
Reindeer	Microbacteriaceae	Family	X-SEZC	Duodenum	3.778	3.422	0.0314	Bacteria > Actinomycetota > Actinobacteria > Micrococcales > Microbacteriaceae
Reindeer	Porphyromonadaceae	Family	X-SEZC	Duodenum	3.576	3.272	0.0415	Bacteria > Bacteroidota > Bacteroidia > Bacteroidales > Porphyromonadaceae
Reindeer	*Porphyromonas*	Genus	X-SEZC	Duodenum	3.576	3.252	0.0415	Bacteria > Bacteroidota > Bacteroidia > Bacteroidales > Porphyromonadaceae > Porphyromonas
Reindeer	Terriglobales	Order	X-SEZC	Duodenum	3.269	2.917	0.0240	Bacteria > Acidobacteriota > Acidobacteriae > Terriglobales
Reindeer	Ktedonobacterales	Order	X-SEZC	Duodenum	2.668	2.858	0.0460	Bacteria > Chloroflexota > Ktedonobacteria > Ktedonobacterales
Reindeer	Ktedonobacteria	Class	X-SEZC	Duodenum	2.668	2.853	0.0460	Bacteria > Chloroflexota > Ktedonobacteria
Reindeer	Ktedonobacteraceae	Family	X-SEZC	Duodenum	2.668	2.828	0.0460	Bacteria > Chloroflexota > Ktedonobacteria > Ktedonobacterales > Ktedonobacteraceae
Reindeer	Coriobacteriia	Class	X-WW	Reticulum	5.188	4.799	0.0465	Bacteria > Actinomycetota > Coriobacteriia
Reindeer	Coriobacteriales	Order	X-WW	Reticulum	5.188	4.779	0.0465	Bacteria > Actinomycetota > Coriobacteriia > Coriobacteriales
Reindeer	*Gordonibacter*	Genus	X-WW	Reticulum	5.116	4.724	0.0333	Bacteria > Actinomycetota > Coriobacteriia > Coriobacteriales > Eggerthellaceae > Gordonibacter
Reindeer	Williamwhitmaniaceae	Family	X-WW	Reticulum	4.129	3.793	0.0069	Bacteria > Bacteroidota > Bacteroidia > Bacteroidales > Williamwhitmaniaceae
Reindeer	Actinomycetaceae	Family	X-WW	Reticulum	4.154	3.772	0.0372	Bacteria > Actinomycetota > Actinobacteria > Actinomycetales > Actinomycetaceae
Reindeer	Actinomycetales	Order	X-WW	Reticulum	4.154	3.770	0.0372	Bacteria > Actinomycetota > Actinobacteria > Actinomycetales
Reindeer	*Actinotignum sp 313*	Species	X-WW	Reticulum	3.938	3.568	0.0159	Bacteria > Actinomycetota > Actinobacteria > Actinomycetales > Actinomycetaceae > Actinotignum > Actinotignum sp 313
Reindeer	Bacillota	Phylum	X-WW	Reticulum	3.845	3.453	0.0110	Bacteria > Bacillota
Reindeer	*Desulfovibrio fairfieldensis*	Species	X-WW	Reticulum	3.811	3.445	0.0377	Bacteria > Thermodesulfobacteriota > Desulfovibrionia > Desulfovibrionales > Desulfovibrionaceae > Desulfovibrio > Desulfovibrio fairfieldensis
Reindeer	Carnobacteriaceae	Family	X-WW	Reticulum	3.652	3.369	0.0345	Bacteria > Bacillota > Bacilli > Lactobacillales > Carnobacteriaceae
Reindeer	Oscillospirales	Order	X-WW	Reticulum	3.484	3.041	0.0164	Bacteria > Bacillota > Clostridia > Oscillospirales
Reindeer	*Howardella*	Genus	X-WW	Reticulum	3.166	2.988	0.0394	Bacteria > Bacillota > Clostridia > Lachnospirales > Lachnospiraceae > Howardella
Reindeer	*Escherichia coli*	Species	X-ZC	Rectum	5.663	5.333	0.0462	Bacteria > Pseudomonadota > Gammaproteobacteria > Enterobacterales > Enterobacteriaceae > Escherichia Shigella > Escherichia coli
Reindeer	Enterobacterales	Order	X-ZC	Rectum	5.668	5.328	0.0492	Bacteria > Pseudomonadota > Gammaproteobacteria > Enterobacterales
Reindeer	Pseudomonadota	Phylum	X-ZC	Rectum	5.698	5.322	0.0170	Bacteria > Pseudomonadota
Reindeer	*Intestinimonas*	Genus	X-ZC	Rectum	3.664	3.333	0.0251	Bacteria > Bacillota > Clostridia > Oscillospirales > Oscillospiraceae > Intestinimonas
Reindeer	*Denitrobacterium detoxificans*	Species	X-ZC	Rectum	3.110	3.005	0.0450	Bacteria > Actinomycetota > Coriobacteriia > Coriobacteriales > Eggerthellaceae > Denitrobacterium > Denitrobacterium detoxificans
Reindeer	*Escherichia Shigella*	Genus	X-ZC	Rectum	3.138	2.986	0.0072	Bacteria > Pseudomonadota > Gammaproteobacteria > Enterobacterales > Enterobacteriaceae > Escherichia Shigella
Reindeer	Mycoplasmatales	Order	X-ZW	Abomasum	5.842	5.425	0.0052	Bacteria > Bacillota > Bacilli > Mycoplasmatales
Reindeer	Mycoplasmataceae	Family	X-ZW	Abomasum	5.842	5.423	0.0052	Bacteria > Bacillota > Bacilli > Mycoplasmatales > Mycoplasmataceae
Reindeer	*Mycoplasmoides*	Genus	X-ZW	Abomasum	5.842	5.403	0.0058	Bacteria > Bacillota > Bacilli > Mycoplasmatales > Mycoplasmataceae > Mycoplasmoides
Reindeer	Actinomycetota	Phylum	X-ZW	Abomasum	5.620	5.191	0.0344	Bacteria > Actinomycetota
Reindeer	*Aeriscardovia*	Genus	X-ZW	Abomasum	5.562	5.178	0.0024	Bacteria > Actinomycetota > Actinobacteria > Bifidobacteriales > Bifidobacteriaceae > Aeriscardovia
Reindeer	Bifidobacteriaceae	Family	X-ZW	Abomasum	5.564	5.152	0.0025	Bacteria > Actinomycetota > Actinobacteria > Bifidobacteriales > Bifidobacteriaceae
Reindeer	Bifidobacteriales	Order	X-ZW	Abomasum	5.564	5.109	0.0025	Bacteria > Actinomycetota > Actinobacteria > Bifidobacteriales
Reindeer	Spirochaetales	Order	X-ZW	Abomasum	5.077	4.725	0.0188	Bacteria > Spirochaetota > Spirochaetia > Spirochaetales
Reindeer	Spirochaetia	Class	X-ZW	Abomasum	5.077	4.723	0.0188	Bacteria > Spirochaetota > Spirochaetia
Reindeer	Spirochaetota	Phylum	X-ZW	Abomasum	5.077	4.718	0.0188	Bacteria > Spirochaetota
Reindeer	Clostridia	Class	X-ZW	Abomasum	5.060	4.629	0.0107	Bacteria > Bacillota > Clostridia
Reindeer	Moraxellaceae	Family	X-ZW	Abomasum	4.691	4.473	0.0066	Bacteria > Pseudomonadota > Gammaproteobacteria > Pseudomonadales > Moraxellaceae
Reindeer	*Acinetobacter*	Genus	X-ZW	Abomasum	4.688	4.464	0.0035	Bacteria > Pseudomonadota > Gammaproteobacteria > Pseudomonadales > Moraxellaceae > Acinetobacter
Reindeer	Pseudomonadales	Order	X-ZW	Abomasum	4.696	4.452	0.0083	Bacteria > Pseudomonadota > Gammaproteobacteria > Pseudomonadales
Reindeer	*Treponema*	Genus	X-ZW	Abomasum	4.603	4.293	0.0447	Bacteria > Spirochaetota > Spirochaetia > Spirochaetales > Spirochaetaceae > Treponema
Reindeer	Negativicutes	Class	X-ZW	Abomasum	4.573	4.135	0.0233	Bacteria > Bacillota > Negativicutes
Reindeer	*Vibrionimonas*	Genus	X-ZW	Abomasum	4.476	4.071	0.0405	Bacteria > Bacteroidota > Bacteroidia > Chitinophagales > Chitinophagaceae > Vibrionimonas
Reindeer	Acidaminococcales	Order	X-ZW	Abomasum	4.255	3.910	0.0043	Bacteria > Bacillota > Negativicutes > Acidaminococcales
Reindeer	*Succiniclasticum*	Genus	X-ZW	Abomasum	4.254	3.883	0.0039	Bacteria > Bacillota > Negativicutes > Acidaminococcales > Acidaminococcaceae > Succiniclasticum
Reindeer	Acidaminococcaceae	Family	X-ZW	Abomasum	4.255	3.874	0.0043	Bacteria > Bacillota > Negativicutes > Acidaminococcales > Acidaminococcaceae
Reindeer	Veillonellales Selenomonadales	Order	X-ZW	Abomasum	4.289	3.831	0.0390	Bacteria > Bacillota > Negativicutes > Veillonellales Selenomonadales
Reindeer	hoa5 07d05 gut group	Genus	X-ZW	Abomasum	4.249	3.754	0.0100	Bacteria > Bacteroidota > Bacteroidia > Bacteroidales > Rikenellaceae > hoa5 07d05 gut group
Reindeer	*Burkholderia Caballeronia Paraburkholderia*	Genus	X-ZW	Abomasum	4.112	3.651	0.0483	Bacteria > Pseudomonadota > Gammaproteobacteria > Burkholderiales > Burkholderiaceae > Burkholderia Caballeronia Paraburkholderia
Reindeer	Acetobacterales	Order	X-ZW	Abomasum	4.049	3.620	0.0148	Bacteria > Pseudomonadota > Alphaproteobacteria > Acetobacterales
Reindeer	Cyanobacteriota	Phylum	X-ZW	Abomasum	4.008	3.615	0.0256	Bacteria > Cyanobacteriota
Reindeer	Vampirivibrionia	Class	X-ZW	Abomasum	3.923	3.556	0.0169	Bacteria > Cyanobacteriota > Vampirivibrionia
Reindeer	*Xylanibacter*	Genus	X-ZW	Abomasum	3.858	3.489	0.0168	Bacteria > Bacteroidota > Bacteroidia > Bacteroidales > Prevotellaceae > Xylanibacter
Reindeer	Synergistia	Class	X-ZW	Abomasum	3.758	3.429	0.0021	Bacteria > Synergistota > Synergistia

Species	Taxon	Taxonomic level	Enriched group	Segment	Original LEfSe abundance score	LDA score (log10)	P value	Full taxonomy
Reindeer	Synergistaceae	Family	X-ZW	Abomasum	3.758	3.423	0.0021	Bacteria > Synergistota > Synergistia > Synergistales > Synergistaceae
Reindeer	Anaerolineae	Class	X-ZW	Abomasum	3.725	3.419	0.0058	Bacteria > Chloroflexota > Anaerolineae
Reindeer	Fibrobacteraceae	Family	X-ZW	Abomasum	3.830	3.418	0.0248	Bacteria > Fibrobacterota > Fibrobacteria > Fibrobacterales > Fibrobacteraceae
Reindeer	Fibrobacterota	Phylum	X-ZW	Abomasum	3.830	3.411	0.0248	Bacteria > Fibrobacterota
Reindeer	Synergistales	Order	X-ZW	Abomasum	3.758	3.409	0.0021	Bacteria > Synergistota > Synergistia > Synergistales
Reindeer	Fibrobacteria	Class	X-ZW	Abomasum	3.830	3.408	0.0248	Bacteria > Fibrobacterota > Fibrobacteria
Reindeer	Synergistota	Phylum	X-ZW	Abomasum	3.758	3.407	0.0021	Bacteria > Synergistota
Reindeer	Fibrobacterales	Order	X-ZW	Abomasum	3.830	3.397	0.0248	Bacteria > Fibrobacterota > Fibrobacteria > Fibrobacterales
Reindeer	Anaerolineaceae	Family	X-ZW	Abomasum	3.718	3.384	0.0047	Bacteria > Chloroflexota > Anaerolineae > Anaerolineales > Anaerolineaceae
Reindeer	Anaerolineales	Order	X-ZW	Abomasum	3.718	3.371	0.0047	Bacteria > Chloroflexota > Anaerolineae > Anaerolineales
Reindeer	*Flexilinea*	Genus	X-ZW	Abomasum	3.718	3.367	0.0039	Bacteria > Chloroflexota > Anaerolineae > Anaerolineales > Anaerolineaceae > Flexilinea
Reindeer	Elusimicrobiota	Phylum	X-ZW	Abomasum	3.623	3.331	0.0209	Bacteria > Elusimicrobiota
Reindeer	*Quinella*	Genus	X-ZW	Abomasum	3.736	3.329	0.0069	Bacteria > Bacillota > Negativicutes > Veillonellales Selenomonadales > Selenomonadaceae > Quinella
Reindeer	*Fibrobacter*	Genus	X-ZW	Abomasum	3.718	3.291	0.0241	Bacteria > Fibrobacterota > Fibrobacteria > Fibrobacterales > Fibrobacteraceae > Fibrobacter
Reindeer	*Mailhella*	Genus	X-ZW	Abomasum	3.668	3.285	0.0075	Bacteria > Thermodesulfobacteriota > Desulfovibrionia > Desulfovibrionales > Desulfovibrionaceae > Mailhella
Reindeer	Endomicrobiales	Order	X-ZW	Abomasum	3.532	3.257	0.0182	Bacteria > Elusimicrobiota > Endomicrobiia > Endomicrobiales
Reindeer	Endomicrobiia	Class	X-ZW	Abomasum	3.532	3.255	0.0182	Bacteria > Elusimicrobiota > Endomicrobiia
Reindeer	*Pyramidobacter*	Genus	X-ZW	Abomasum	3.639	3.254	0.0090	Bacteria > Synergistota > Synergistia > Synergistales > Synergistaceae > Pyramidobacter
Reindeer	*Endomicrobium*	Genus	X-ZW	Abomasum	3.532	3.247	0.0182	Bacteria > Elusimicrobiota > Endomicrobiia > Endomicrobiales > Endomicrobiaceae > Endomicrobium
Reindeer	Endomicrobiaceae	Family	X-ZW	Abomasum	3.532	3.227	0.0182	Bacteria > Elusimicrobiota > Endomicrobiia > Endomicrobiales > Endomicrobiaceae
Reindeer	Prolixibacteraceae	Family	X-ZW	Abomasum	3.447	3.190	0.0467	Bacteria > Bacteroidota > Bacteroidia > Bacteroidales > Prolixibacteraceae
Reindeer	*Bifidobacterium*	Genus	X-ZW	Abomasum	3.151	3.182	0.0270	Bacteria > Actinomycetota > Actinobacteria > Bifidobacteriales > Bifidobacteriaceae > Bifidobacterium
Reindeer	Bacteroides pectinophilus group	Genus	X-ZW	Abomasum	3.228	3.083	0.0289	Bacteria > Bacillota > Clostridia > Lachnospirales > Lachnospiraceae > Bacteroides pectinophilus group
Reindeer	*Succinivibrio*	Genus	X-ZW	Abomasum	3.137	3.074	0.0481	Bacteria > Pseudomonadota > Gammaproteobacteria > Enterobacterales > Succinivibrionaceae > Succinivibrio
Reindeer	*Mangroviflexus*	Genus	X-ZW	Abomasum	3.462	3.046	0.0359	Bacteria > Bacteroidota > Bacteroidia > Bacteroidales > Marinilabiliaceae > Mangroviflexus
Reindeer	CAG 352	Genus	X-ZW	Abomasum	3.385	3.040	0.0278	Bacteria > Bacillota > Clostridia > Oscillospirales > Ruminococcaceae > CAG 352
Reindeer	Marinilabiliaceae	Family	X-ZW	Abomasum	3.462	3.039	0.0359	Bacteria > Bacteroidota > Bacteroidia > Bacteroidales > Marinilabiliaceae
Reindeer	Desulfuromonadia	Class	X-ZW	Abomasum	3.352	2.970	0.0410	Bacteria > Thermodesulfobacteriota > Desulfuromonadia
Reindeer	*Ruminococcus albus*	Species	X-ZW	Abomasum	3.152	2.951	0.0215	Bacteria > Bacillota > Clostridia > Oscillospirales > Ruminococcaceae > Ruminococcus > Ruminococcus albus
Reindeer	*Pseudolabrys taiwanensis*	Species	X-ZW	Abomasum	3.288	2.892	0.0229	Bacteria > Pseudomonadota > Alphaproteobacteria > Hyphomicrobiales > Xanthobacteraceae > Pseudolabrys > Pseudolabrys taiwanensis
