# Supplementary material for: Comparative Analysis of Gastrointestinal Microbiota Along the Digestive Tract in Sika Deer and Reindeer and Prediction of Their Potential Function
Source: Animals (Basel). 2026 May 11;16(10):1476. doi: 10.3390/ani16101476 (PMC13203257; doi:10.3390/ani16101476)
Supplement: Supplementary file 1 [file animals-16-01476-s001.zip › Supplementary Materials TableS1-S2.pdf]

Supplementary Materials:

Table S1: Alpha diversity indices of microbiota across gastrointestinal segments in reindeer and sika deer

Species	Gastrointestinal Segment	n	Observed-species	Chao1	Shannon	Simpson
Reindeer	Rumen	3	1905.67 ± 48.34	2363.77 ± 49.44	5.46 ± 0.25	0.98 ± 0.01
Reindeer	Reticulum	3	1755.33 ± 62.55	2266.55 ± 89.81	4.72 ± 0.15	0.94 ± 0.01
Reindeer	Omasum	3	1776.67 ± 39.32	2283.60 ± 159.86	4.42 ± 0.23	0.90 ± 0.03
Reindeer	Abomasum	3	1925.33 ± 112.51	2374.26 ± 86.02	5.12 ± 0.23	0.96 ± 0.00
Reindeer	Duodenum	3	1728.33 ± 226.39	2231.55 ± 232.78	4.43 ± 0.64	0.89 ± 0.06
Reindeer	Jejunum	3	1452.00 ± 244.22	1933.71 ± 268.21	3.94 ± 0.44	0.87 ± 0.04
Reindeer	Cecum	3	1825.33 ± 287.59	2318.85 ± 223.02	4.82 ± 0.75	0.91 ± 0.07
Reindeer	Colon	3	1615.00 ± 301.43	2141.55 ± 348.63	4.28 ± 1.04	0.86 ± 0.10
Reindeer	Rectum	3	1523.00 ± 253.75	1967.94 ± 304.23	3.88 ± 0.53	0.87 ± 0.04
Sika deer	Rumen	3	1717.33 ± 980.29	2363.44 ± 1298.05	5.15 ± 1.25	0.96 ± 0.04
Sika deer	Reticulum	3	1575.67 ± 1127.87	2146.64 ± 1505.11	5.23 ± 1.36	0.96 ± 0.04
Sika deer	Omasum	3	3107.67 ± 335.51	4383.59 ± 546.30	6.24 ± 0.18	0.99 ± 0.01
Sika deer	Abomasum	3	3050.00 ± 464.68	4278.40 ± 781.52	6.00 ± 0.18	0.98 ± 0.00
Sika deer	Duodenum	3	2293.33 ± 421.25	3176.42 ± 603.00	6.05 ± 0.50	0.99 ± 0.01
Sika deer	Jejunum	3	1316.67 ± 307.74	1884.98 ± 667.60	4.87 ± 0.89	0.96 ± 0.05
Sika deer	Ileum	3	2882.33 ± 1459.04	3922.82 ± 1850.56	5.30 ± 1.95	0.90 ± 0.15
Sika deer	Cecum	3	2537.67 ± 1210.77	3602.85 ± 1869.05	5.28 ± 1.05	0.96 ± 0.02
Sika deer	Colon	3	3423.33 ± 683.20	4908.76 ± 1238.33	6.40 ± 0.42	0.99 ± 0.01
Sika deer	Rectum	3	3599.00 ± 787.69	5102.04 ± 1357.69	6.57 ± 0.16	0.99 ± 0.00

Table S2. Student's t-test results for alpha diversity indices between reindeer and sika deer in shared gastrointestinal segments

Gastrointestinal Segment	Index	Reindeer	Sika deer	T value	P value
Rumen	Observed-species	1905.67 ± 48.34	1717.33 ± 980.29	0.332	0.7563
Rumen	Chao1	2363.77 ± 49.44	2363.44 ± 1298.05	0	0.9997
Rumen	Shannon	5.46 ± 0.25	5.15 ± 1.25	0.422	0.6946
Rumen	Simpson	0.98 ± 0.01	0.96 ± 0.04	0.719	0.5121
Reticulum	Observed-species	1755.33 ± 62.55	1575.67 ± 1127.87	0.275	0.7966
Reticulum	Chao1	2266.55 ± 89.81	2146.64 ± 1505.11	0.138	0.8971
Reticulum	Shannon	4.72 ± 0.15	5.23 ± 1.36	-0.638	0.558
Reticulum	Simpson	0.94 ± 0.01	0.96 ± 0.04	-0.925	0.4073
Omasum	Observed-species	1776.67 ± 39.32	3107.67 ± 335.51	-6.824	0.0024
Omasum	Chao1	2283.60 ± 159.86	4383.59 ± 546.30	-6.39	0.0031
Omasum	Shannon	4.42 ± 0.23	6.24 ± 0.18	-10.878	0.0004
Omasum	Simpson	0.90 ± 0.03	0.99 ± 0.01	-5.555	0.0051
Abomasum	Observed-species	1925.33 ± 112.51	3050.00 ± 464.68	-4.074	0.0152
Abomasum	Chao1	2374.26 ± 86.02	4278.40 ± 781.52	-4.195	0.0138
Abomasum	Shannon	5.12 ± 0.23	6.00 ± 0.18	-5.178	0.0066
Abomasum	Simpson	0.96 ± 0.00	0.98 ± 0.00	-4.797	0.0087
Duodenum	Observed-species	1728.33 ± 226.39	2293.33 ± 421.25	-2.046	0.1102
Duodenum	Chao1	2231.55 ± 232.78	3176.42 ± 603.00	-2.532	0.0645
Duodenum	Shannon	4.43 ± 0.64	6.05 ± 0.50	-3.47	0.0256
Duodenum	Simpson	0.89 ± 0.06	0.99 ± 0.01	-2.848	0.0465
Jejunum	Observed-species	1452.00 ± 244.22	1316.67 ± 307.52	0.597	0.5827
Jejunum	Chao1	1933.71 ± 268.21	1884.98 ± 667.60	0.117	0.9123
Jejunum	Shannon	3.94 ± 0.44	4.87 ± 0.89	-1.616	0.1814
Jejunum	Simpson	0.87 ± 0.04	0.96 ± 0.05	-2.297	0.0833
Cecum	Observed-species	1825.33 ± 287.59	2537.67 ± 1211.86	-0.991	0.378
Cecum	Chao1	2318.85 ± 223.02	3602.85 ± 1869.05	-1.182	0.3028
Cecum	Shannon	4.82 ± 0.75	5.28 ± 1.05	-0.617	0.5706
Cecum	Simpson	0.91 ± 0.07	0.96 ± 0.02	-1.232	0.2853
Colon	Observed-species	1615.00 ± 301.43	3423.33 ± 683.07	-4.195	0.0138
Colon	Chao1	2141.55 ± 348.63	4908.76 ± 1238.33	-3.726	0.0204
Colon	Shannon	4.28 ± 1.04	6.40 ± 0.42	-3.288	0.0303
Colon	Simpson	0.86 ± 0.10	0.99 ± 0.01	-2.145	0.0986
Rectum	Observed-species	1523.00 ± 253.75	3599.00 ± 735.02	-4.624	0.0098
Rectum	Chao1	1967.94 ± 304.23	5102.04 ± 1357.69	-3.902	0.0175
Rectum	Shannon	3.88 ± 0.53	6.57 ± 0.16	-8.489	0.0011
Rectum	Simpson	0.87 ± 0.04	0.99 ± 0.00	-5.112	0.0069

Values are presented as mean ± SD. Student's t-test was used to compare alpha diversity indices between reindeer and sika deer within the same gastrointestinal segment. Because reindeer ileal samples were not available, the ileum was excluded from direct interspecific comparisons. $P < 0.05$ was considered statistically significant.
